# *Bacillus subtilis* biofilm extends *Caenorhabditis elegans* longevity through downregulation of the insulin-like signalling pathway

**DOI:** 10.1038/ncomms14332

**Published:** 2017-01-30

**Authors:** Verónica Donato, Facundo Rodríguez Ayala, Sebastián Cogliati, Carlos Bauman, Juan Gabriel Costa, Cecilia Leñini, Roberto Grau

**Affiliations:** 1 Departamento de Microbiología, Facultad de Ciencias Bioquímicas y Farmacéuticas, Universidad Nacional de Rosario, CONICET, Rosario 2000, Argentina

## Abstract

Beneficial bacteria have been shown to affect host longevity, but the molecular mechanisms mediating such effects remain largely unclear. Here we show that formation of *Bacillus subtilis* biofilms increases *Caenorhabditis elegans* lifespan. Biofilm-proficient *B. subtilis* colonizes the *C. elegans* gut and extends worm lifespan more than biofilm-deficient isogenic strains. Two molecules produced by *B. subtilis* — the quorum-sensing pentapeptide CSF and nitric oxide (NO) — are sufficient to extend *C. elegans* longevity. When *B. subtilis* is cultured under biofilm-supporting conditions, the synthesis of NO and CSF is increased in comparison with their production under planktonic growth conditions. We further show that the prolongevity effect of *B. subtilis* biofilms depends on the DAF-2/DAF-16/HSF-1 signalling axis and the downregulation of the insulin-like signalling (ILS) pathway.

## Abstract (web summary)

Probiotic bacteria can improve host health, but the mechanisms underlying such beneficial effects are often unclear. Here, the authors show that biofilm formation of the probiotic bacterium *B. subtilis* extends the lifespan of its host, the nematode *C. elegans*, by reducing insulin-like signalling.

During the last 30 years, human life expectancy has significantly increased worldwide http://www.who.int/gho/publications/world_health_statistics/2015. Between 1990 and 2012, humans (males and females) born in low- and high-income countries have gained 9.05 and 4.25 years in life expectancy, respectively http://www.who.int/gho/publications/world_health_statistics/2015, which has been attributed to a plethora of genetic and environmental factors^1^,^2^. In mammals, the beneficial bacteria residing in the host intestine (that is, gut or commensal flora) play a crucial role in immune system development, tissue morphogenesis and aging^3^,^4^,^5^,^6^,^7^,^8^. Beneficial effects of the gut flora on host physiology, including slowing of aging, might be improved with the incorporation of probiotic bacteria into the host diet. Probiotics are live organisms that have a beneficial effect on host health when they are administered or present in adequate quantities^9^,^10^. However, the mechanisms causing potential prolongevity effects of beneficial bacteria on different hosts remain poorly understood^10^,^11^,^12^,^13^,^14^,^15^.

Microbial biofilms are three-dimensional structured communities of adherent microorganisms encased in a self-produced extracellular matrix, containing networks of channels for nutrient supply and long-distance cell-to-cell communication^16^,^17^. Bacteria living in biofilms are physiological very distinct from their planktonic counterparts, and they function as a cooperative consortium more similar to that of multicellular organisms than a unicellular organism^16^,^18^,^19^. The crucial role of biofilms in the success of different pathogenic bacteria in infecting different hosts is well documented^20^, but whether biofilms influence the interaction between beneficial bacteria and their host is largely unknown^10^,^16^.

*Bacillus subtilis* is a model beneficial bacterium with the ability to display many distinct cell types under developmental control, including the ability to form robust and sophisticated biofilms^21^,^22^,^23^,^24^,^25^,^26^,^27^. Interestingly, a recent study showed that planktonic *B. subtilis* modulates the longevity of the bacteriovorus nematode and model organism *Caenorhabditis elegans* independent of its role as a food source^21^,^28^. Worms fed on planktonic *B. subtilis* cells live longer (an approximately 15% increase in lifespan) than worms grown in the presence of other food sources, that is, *E. coli*^28^,^29^. Because of the limited understanding of the effect of biofilm formed by beneficial gut bacteria on host health, we determined whether the biofilm of *B. subtilis* might represent a novel anti-aging agent for improving host longevity.

## Results and Discussion

### *C. elegans* longevity is increased by undomesticated *B. subtilis*

The various studies that have reported interactions of *B. subtilis* with *C. elegans* utilized genetically modified laboratory *B. subtilis* (domesticated) strains derived from the wild (undomesticated) Marburg isolate NCIB3610. However, these laboratory strains (that is, strains 168, JH642 and PY79; refs 30, 31, 32 are known to harbour several initially inadvertent mutations that affect collective behaviours (that is, social-surface motility and biofilm formation)^31^,^32^ that might play an important role during bacteria–host interactions.

**Figure 1:**
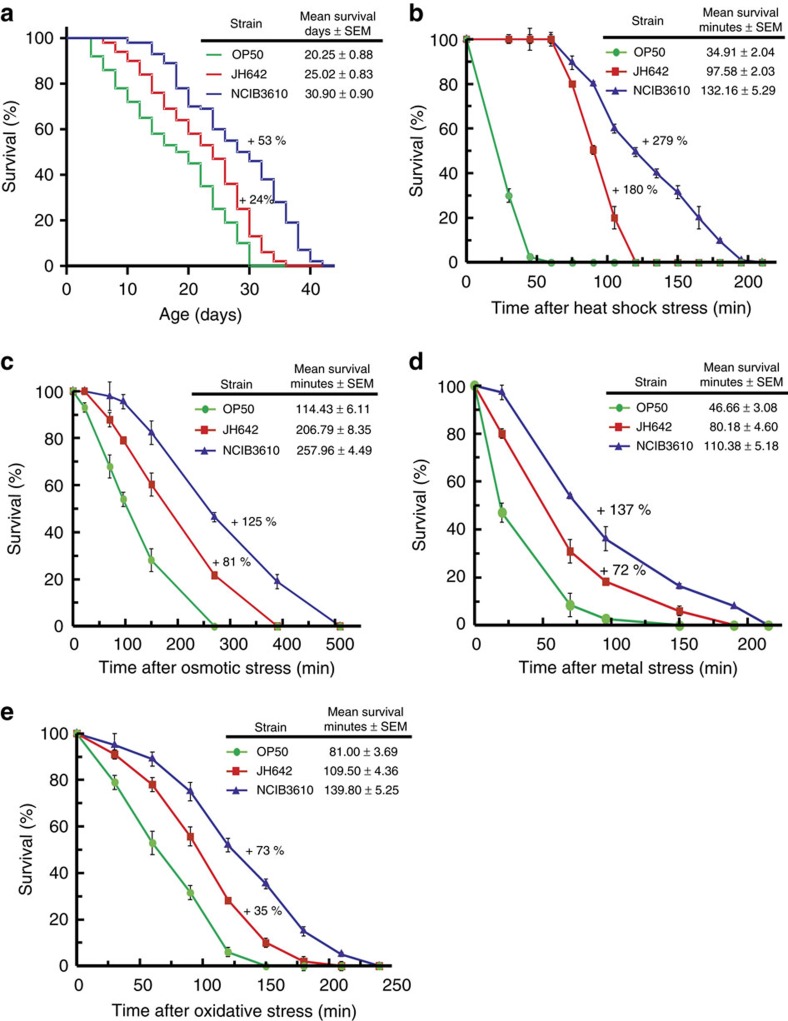
Undomesticated *B. subtilis* extends *C. elegans* survival. (**a**) *C. elegans* longevity fed on *B. subtilis* cells. Fifty late L4/young-adult-stage N2 worms were fed either on wild-type spores of domesticated JH642 (red) or undomesticated NCIB3610 (blue) *B. subtilis* on NGM agar plates, and survival was monitored. (**b**) Thermotolerance of *C. elegans* fed on *B. subtilis*. Thirty late L4/young-adult-stage worms on NGM plates fed either on *B. subtilis* JH642 (red) or NCIB3610 (blue) on NGM plates were shifted from 20 to 34 °C, and the mean survival was calculated. (**c**–**e**) Effects of different stresses on *C. elegans* survival. Thirty late L4/young-adult-stage worms were fed either on wild-type spores of domesticated JH642 (red) or undomesticated NCIB3610 (blue) *B. subtilis* on NGM agar plates, transferred to 24-well plates containing 200 mM NaCl (for osmotic stress, **c**), 50 μM cadmium (for metal stress, **d**), 25 mM H_2_O_2_ (for oxidative stress, **e**). At 30 min intervals, the worms were scored for signs of life. OP50 *E. coli* (green) was used as a control. Each graph (**a**–**e**) is the average**±**s.e.m. (*n*=3), and the survival increase is expressed as a percentage of the total number of worms fed on OP50 *E. coli*.

To answer this question, we initially compared the lifespan effects produced by the wild strain NCIB3610 (ref. 31) and its isogenic derivate, the domesticated JH642 strain^33^ (Supplementary Table 1) on *C. elegans*. It has been noted that in contrast to the susceptibility of vegetative cells, *B. subtilis* spores survive the transit through the pharynx and germinate in the worm intestine^34^,^35^. Therefore, for all the subsequent experiments, the sporulated form of *B. subtilis* was provided as the worm food source to correlate the effects on worm lifespan with the activity of live bacteria^10^,^21^. *C. elegans* fed on domesticated *B. subtilis* cells showed an extended longevity compared with the effect of OP50 *E. coli* cells when used as a food source (Fig. 1a and Supplementary Table 2). The survival of worms fed on JH642 cells increased by an average of 23.56% (average survival, 25.02 days; 95% confidence interval (CI): 23.39–26.65) compared with strain OP50 (average survival, 20.25 days; 95% CI: 18.52–21.97) when used as a food source (Supplementary Table 2). Interestingly, the lifespan effect on *C. elegans* was significantly more pronounced with a statistically right-shifted survival curve (*P*=0.00) by log-rank test when the worms were fed on undomesticated *B. subtilis* cells (strain NCIB3610) (Fig. 1a and Supplementary Table 2). In this case, NCIB3610 cells increased the worm longevity by an average of 52.59% compared with the lifespan effect produced by OP50 *E. coli* cells (average value, 30.90 days; 95% CI: 29.14–32.67) (Supplementary Table 2). The lifespan of *C. elegans* also increased by an average of 59.51% (average value, 32.30 days; 95% CI: 30.68–33.90; equality, *P*=0.00 by log-rank test) when fed on another undomesticated *B. subtilis* strain (that is, the probiotic *B. subtilis* natto strain RG4365, Supplementary Table 1)^22^,^27^. The different effects of these bacterial strains on worm survival cannot be attributed to physiological differences between the domesticated and undomesticated strains because both types of bacterial cells displayed similar efficiencies of spore germination, vegetative growth and spore formation (Supplementary Fig. 1a–c). However, the possibility exists that the observed differential effects on worm survival were due to changes in the mechanical/physical properties of both types of spores. In this case, the worm grinder may not be able to efficiently break the spores of undomesticated cells, leading to higher colonization levels and/or reduced nutritional availability, which might explain the observed longevity effects (Fig. 1a). To test this possibility, we analysed and compared the robustness and resistance of JH642 and NCIB3610 spores. As shown in Supplementary Fig. 1d, spores derived from domesticated and undomesticated cells are indistinguishable one from the other in their resistance to different physico-chemical treatments such as sonication, low and high pH exposure, and lysozyme and protease treatments. Accordingly, several physiological parameters of *C. elegans*, such as its chemotactic behaviour (in response to the different bacterial food sources), growth rate, body size, postembryonic development and egg-laying proficiency were not affected when fed on any of the selected bacterial strains (that is, OP50, JH642 and NCIB3610 cells; Supplementary Fig. 1e–i). Additionally, we used FUdR in the lifespan assays which was previously shown to be a potential co-founder for *C. elegans* lifespan studies^36^. To test this possibility, we analysed the lifespan effects produced by *B. subtilis* NCIB3610 cells on N2 worms either in the presence or the absence of FUdR, as well as the effect of FUdR in *B. subtilis* biofilm formation proficiency. As shown in Supplementary Fig. 2a, and Supplementary Table 3, the worm lifespan increase produced by *B. subtilis* NCIB3610 cells was essentially the same in the presence (average value, 30.90 days; 95% CI: 29.14–32.67; equality, *P*=0.00 by log-rank test) or absence of FUdR (average value, 31.07; 95% CI: 29.45–32.78). Accordingly, the *in vitro* and *in vivo* biofilm formation proficiency of *B. subtilis* NCIB3610 cells was not affected by FUdR (Supplementary Fig. 2b). Overall, these results rule out potential drug–bacterial strain interaction, variations in food preference and/or food availability between the different bacterial food sources as reasons for the observed differences in *C. elegans* lifespan (Fig. 1a and Supplementary Table 2).

Lifespan and stress resistance are interrelated^1^,^2^. Extended longevity of *C. elegans* correlates with enhanced worm resistance against different biological, physical and chemical stressors^37^,^38^. In this sense, in accordance with the increase in the lifespan effect produced by undomesticated *B. subtilis*, we found a strong positive effect on the thermotolerance of *C. elegans*. As shown in Fig. 1b, *C. elegans* fed on NCIB3610 cells showed higher resistance and survival to heat shock compared with the protective effects produced by domesticated JH642 (using OP50 cells as a control). When *C. elegans* was shifted from its routine growth temperature of 20 to 34 °C, worms fed either on undomesticated or domesticated *B. subtilis* cells lived 278.57% (average survival, 132.16 min) and 179.52% (average survival, 97.58 min) longer, respectively, than did worms fed on OP50 cells (average survival, 34.91 min; Fig. 1b and Supplementary Table 2). Accordingly, we did not observe any significant FUdR interference on the thermotolerance behaviour of *C. elegans* when fed on NCIB3610 cells (Supplementary Fig. 2c and Supplementary Table 3).

To confirm the higher beneficial effects of undomesticated *B. subtilis* cells over domesticated *B. subtilis* cells on worm survival, we measured the lifespan of N2 worms subjected to injuries other than heat shock (that is, osmotic, heavy metal and oxidative stresses). In all the analysed situations, *C. elegans* fed on NCIB3610 *B. subtilis* showed a significant increase in survival (equality, *P*<0.001 by log-rank test) compared with the survival of worms fed on JH642 or OP50 cells (Fig. 1c–e). Taken together, these results indicate that wild (undomesticated) *B. subtilis* strains are significantly more beneficial and efficient than domesticated *B. subtilis* in protecting and prolonging *C. elegans* lifespan (Fig. 1, Table 1A and Supplementary Table 2).

### *C. elegans* longevity depends on *B. subtilis* gut colonization

**Figure 2:**
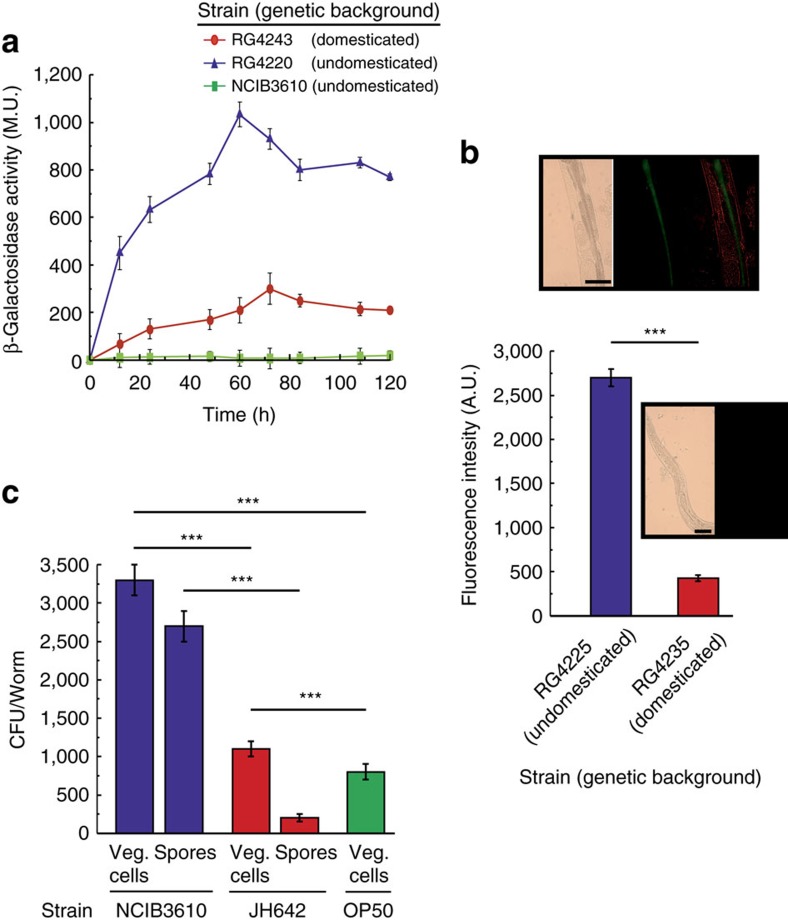
Proficiency of *B. subtilis* gut colonization in *C. elegans* intestine. (**a**) Expression of surfactin operon in worm intestine. Thirty late L4/young-adult-stage worms were allowed to develop on NGM agar plates seeded with OP50 *E. coli*, washed and transferred to fresh NGM plates containing JH642- or NCIB3610-derived isogenic RG4243 (red circles) and RG4220 (blue circles) strains, respectively (Supplementary Table 1) harbouring the P_*srf-lacZ*_ reporter gene. Determination of β-galactosidase activity was performed in worm extracts. The β-galactosidase activity, expressed as Miller units (MU) over time (h) is shown. ***P<0.001 (analysis of variance (ANOVA) with Bonferroni test). (**b**) *B. subtilis* biofilm formation in *C. elegans* gut. Thirty late L4/young-adult-stage worms were allowed to develop NGM agar plates seeded with OP50 *E. coli*, washed and transferred to fresh NGM plates containing NCIB3610- or JH642-derived isogenic (RG4225 (blue) and RG4235 (red) strains, respectively, carrying an integrated *bslA::gfp* reporter fusion (Supplementary Table 1). The worms were taken and imaged by fluorescence microscopy. Fluorescence intensity is indicated as arbitrary units (A.U.) per worm, and error bars show the mean**±**(*n*=3) ***P<0.001 (ANOVA with Bonferroni test). The inserts shown are epifluorescence micrographs of typically stained N2 *C. elegans* fed on GFP-expressing domesticated and undomesticated *B. subtilis*. The fluorescence images were superimposed to differential interference contrast (DIC) images to depict the localization of the labels within the cells. Scale bar, 20 μm. (**c**) Worm intestine colonization by *B. subtilis*. Hundred late L4/young-adult-stage worms were allowed to develop on NGM agar plates seeded with OP50 *E. coli*, washed, transferred to fresh NGM plates containing new OP50 cells (green) or spores of the strains JH642 (red) or NCIB3610 (blue). The number of *E. coli* or *B. subtilis* cells (spores and vegetative forms) was measured in the worm gut. Error bars show the mean**±**(*n*=3). ***P<0.001 (ANOVA with Bonferroni test).

To shed light on the reasons for the differences in the magnitude of the lifespan effects produced by domesticated and undomesticated *B. subtilis*, we analysed the ability of each cell type to grow and colonize the worm intestine^14^,^15^,^34^. Our initial approach was to measure the intestinal activity of a reporter gene harboured by the bacterium used as the worm food source. Therefore, we fed *C. elegans* on NCIB3610 and JH642 cells that harboured a reporter of β-galactosidase activity^30^ driven by the bacterium promoter of the surfactin operon (Supplementary Table 1). As shown in Fig. 2a, cellular extracts obtained from worms fed on both types of *B. subtilis* cells showed β-galactosidase activity derived from the reporter p_*srf-lacZ*_ fusion, a finding that strongly suggests that both types of spores (JH642 and NCIB3610) were able to germinate (spores are metabolically inactive) in *C. elegans* gut. Interestingly, the pattern of β-galactosidase expression obtained from the undomesticated cells was significantly higher than that obtained from the worms fed on domesticated cells; peaks of 200 and 1,000 Miller units (MU) of β-galactosidase activity were reached after 70 h of gut colonization with JH642 and NCIB3610 isogenic strains harbouring the *lacZ* reporter-fusion system, respectively (Fig. 2a). The difference in the level of expression of the p_*srf-lacZ*_ reporter fusion could be due to a different spore germination or bacterial growth rate, to the differential expression of the surfactin promoter in domesticated and undomesticated cells and/or to a different level of bacterial gut colonization (number of *B. subtilis* cells/worm). To distinguish between these possibilities, we measured the germination rates, the outgrowth and the β-galactosidase expression driven by the P_*srf-lacZ*_ fusion in domesticated and undomesticated *B. subtilis* cells inoculated in nematode growth medium (NGM). As shown in Supplementary Fig. 3, the kinetics of spore germination, the vegetative growth rates and the β-galactosidase expression from the reporter p_*srf-lacZ*_ fusion of cells grown in liquid and solid NGM did not significantly differ between the domesticated and undomesticated *B. subtilis* cells (Supplementary Fig. 3a–c). These results suggest that the different levels of intestinal β-galactosidase activity derived from the two types of utilized *B. subtilis* cells (Fig. 2a) were due to the different levels of bacterial worm gut colonization.

Similarly, the fluorescence signal originated from the gut of worms fed on undomesticated *B. subtilis* cells harbouring a reporter gene of GFP expression (*bslA-gfp*) (Supplementary Table 1) was significantly higher than the originated from worms fed on domesticated *B. subtilis* cells (Fig. 2b). The observed differences in GFP expression were not due to differences in the growth rate or other physiological parameter between both types of *B. subtilis* strains because the GFP expression levels displayed by both types of cells were indistinguishable from one another under planktonic growth conditions on NGM agar plates (Supplementary Fig. 3d). Therefore, these results demonstrate the proficiency of *B. subtilis*, as a commensal organism, to complete an entire cell cycle in the intestine of *C. elegans* as was previously reported for this bacterium in other animal models^39^,^40^,^41^. In addition, wild *B. subtilis* was hypothesized to be a better colonizer of the worm intestine than domesticated *B. subtilis* (Fig. 2a,b and Supplementary Fig. 3e). Supporting this hypothesis, the number of NCIB3610 cells (spores plus vegetative cells) recovered from the worm gut was significantly higher (4-fold higher) than the intestinal number of JH642 cells (spores plus vegetative cells) (Fig. 2c).

To confirm that undomesticated *B. subtilis* is a better gut colonizer than domesticated *B. subtilis*, we measured the levels of bacterial gut colonization and the lifespan effect on worms in which one bacterial type (NCIB3610 or JH642) used as a food source for the initial colonization (‘bacterial pulse') was switched (‘bacterial chase') with other type of bacteria (OP50, JH642, NCIB3610 or a mixture of them; Supplementary Fig. 4). Overall, the results of the food switching experiments confirmed the higher ability of NCIB3610 cells to colonize and persist in the worm intestine compared with OP50 and domesticated JH642 cells. In addition, each level of worm gut colonization produced by cells of the strains OP50 (Supplementary Fig. 4a,h), JH642 (Supplementary Fig. 4c,i), NCIB3610 (Supplementary Fig. 4b,j) or mixtures of them (Supplementary Fig. 4d–g,k–n) correlates with the lifespan effect produced by these strains when individually used to feed *C. elegans* during its entire lifespan. As observed in Supplementary Fig. 4o,p, the presence of a higher number of NCIB3610 cells in the gut corresponded with the highest worm longevity (Supplementary Table 4). These results demonstrated that wild (undomesticated) *B. subtilis* is better suited to colonize and persist in the intestinal tract of *C. elegans* and to improve its longevity compared with domesticated *B. subtilis* and OP50 cells.

Interestingly, a previous report indicated that only a supply of vegetative *B. subtilis*, and not spores, was able to extend the NO-dependent longevity of *C. elegans*^28^. The failure of the dormant spores to increase the longevity of *C. elegans* was attributed to the inability of these spores to germinate and initiate the *de novo* synthesis of NO^28^. However, our study used sporulated *B. subtilis* that was able to germinate, grow, colonize the worm intestine, thus extending the worm longevity (Fig. 2). Consistent with our results, other reports have previously indicated that *B. subtilis* spores are better suited to survive the transit from the worm pharynx to the intestine than vegetative cells, which are primarily destroyed during this process^34^,^35^,^42^. We attribute this discrepancy with the mentioned work^28^ to the presence of unnoticed genetic differences in the utilized *B. subtilis* strain (that is, differences in the efficiencies of spore germination and/or outgrowth). Supporting this interpretation, the existence of important genetic differences between domesticated *B. subtilis* strains used in different laboratories around the world has been previously reported^32^,^43^.

### The biofilm is key to colonize *C. elegans* and extend its lifespan

The ability to adhere and form a multicellular structure called a pellicle or biofilm constitutes an attribute of bacteria that is key to their success in colonization and persistence in a particular niche^16^,^17^,^18^,^19^,^20^. In studies of host–bacteria interactions, the biofilm formation proficiency of both pathogenic and beneficial bacteria is believed to play a crucial role^44^,^45^,^46^,^47^,^48^,^49^,^50^,^51^,^52^. Because of the significant difference between domesticated and undomesticated *B. subtilis* strains in their biofilm formation proficiency^24^,^31^, we wanted to analyse whether the observed differences in worm longevity and gut colonization produced by *B. subtilis* were due to the different abilities of the bacterial strains to establish a biofilm in the worm intestinal environment. The *tapA-sipW-tasA* and *epsA-G* operons encode for two key components of the extracellular matrix of the biofilm, the TasA protein and the EPS exopolysaccharide, respectively^25^,^26^,^53^. In addition, the *bslA* gene encodes for an essential hydrophobin responsible for the formation of the hydrophobic surface layer that surrounds and protects the biofilm^54^. Therefore, to test our hypothesis, we introduced mutations in *bslA*, *epsG* and *tasA* genes of the undomesticated strain NCIB3610 to obtain biofilm-defective derivates (Supplementary Table 1).

**Figure 3:**
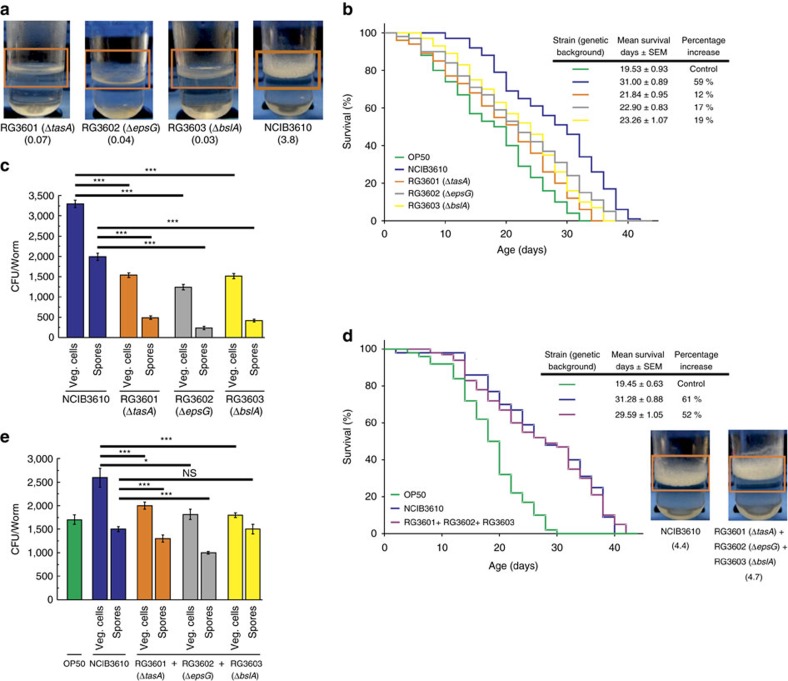
*B subtilis *deficient in biofilm formation cannot extend the longevity of *C. elegans*. (**a**) Biofilm formation proficiency. Biofilms of undomesticated *B. subtilis* strains harbouring mutations in genes encoding for essential components of the biofilm matrix (*ΔepsG*; *ΔtasA* or *ΔbslA*) were developed in liquid NGM. (**b**) Worm longevity is affected when the synthesis of components of the biofilm matrix is reduced. Fifty L4 worms were fed on spores of undomesticated *B. subtilis* deficient in components of the biofilm matrix (*ΔepsG*, grey), (*ΔtasA*, orange) or (*ΔbslA*, yellow) on NGM agar plates, and the survival was monitored. The figure shows the survival average**±**s.e.m. (*n*=3). (**c**) Colonization of the worm gut by *B. subtilis* strains deficient in biofilm formation. Thirty L4 worms were allowed to develop on NGM agar plates seeded with OP50 cells, washed and transferred to fresh NGM plates containing spores of the wild-type NCIB3610 (blue) or spores of each one of the three mutants deficient in biofilm formation. Worms were processed to measure the number of *B. subtilis* cells (spores and vegetative forms) in the worm gut. Error bars show the mean**±**s.e.m. (*n*=3) ***P<0.001 (analysis of variance (ANOVA) with Bonferroni test). (**d**) Transcomplementation of a mixture of biofilm-deficient *B. subtilis* strains to restore *C. elegans* lifespan. Thirty L4 worms were fed either on NCIB3610 (blue) or a mixture of the three *B. subtilis* mutants deficient in biofilm formation (purple) and worm survival was monitored. A representative experiment**±**s.e.m. is shown. (**e**) Recolonization of the worm gut by a proportional mixture of *B. subtilis* strains deficient in biofilm formation. Thirty L4 worms were allowed to develop on NGM agar plates seeded with OP50 cells, washed and transferred to fresh NGM plates containing spores of the wild-type NCIB3610 (blue) or a mixture of the three mutants deficient in biofilm formation. The worms were processed to measure the number of *B. subtilis* cells in the gut worm. Error bars show the mean**±**s.e.m. (*n*=3) ***P<0.001 (ANOVA with Bonferroni test). NS denotes no significance differences between the numbers of *B. subtilis* spores counted.

When each of the three undomesticated-derived *B. subtilis* mutant strains defective in biofilm formation (Δ*tasA*, Δ*epsG* and Δ*bslA*; Fig. 3a) were separately used to feed *C. elegans*, there was a significant decrease in lifespan (an average decrease of 30%) compared with *C. elegans* fed on the biofilm-proficient wild-type strain NCIB3610 (Fig. 3b and Supplementary Table 5). For instance, while *C. elegans* fed on the wild-type NCIB3610 strain showed an increase in longevity of 58.73% (Supplementary Table 5), worms fed on the biofilm-deficient Δ*bslA* strain (RG3603, Supplementary Table 1) only increased longevity by an average of 19.10% (average value, 23.26 days; 95% CI: 21.16–25.36; equality, *P*=0.01 by log-rank test) compared with the lifespan effect produced by OP50 *E. coli* cells (average value, 19.53 days; 95% CI: 17.70–21.36; equality; Supplementary Table 5).

The observed decrease in the worm lifespan was not associated with a diminished ability of the biofilm-mutant strain (Δ*tasA*, Δ*bslA* or Δ*epsG*) spores to germinate and/or to be more or less nutritious for *C. elegans* than the wild-type NCIB3610 cells because we did not observe any differences in the efficiency of spore germination or postembryonic worm development or worm chemotactic behaviour using any of the biofilm mutant strains (Δ*tasA*, Δ*bslA* or Δ*epsG*) compared with NCIB3610 spores as a food source. In addition, the spore robustness against chemical and mechanical injuries was indistinguishable between biofilm-proficient and biofilm-deficient strain spores (data not shown). Importantly, the decrease in worm longevity produced by feeding worms each biofilm-defective strain correlated with a decreased bacterial proficiency in gut colonization (Fig. 3c).

Because EPS and the TasA and BslA proteins are secreted to the extracellular matrix of the biofilm^23^, mixtures of the Δ*bslA*, Δ*eps* and Δ*tasA* mutant cells complement each other to restore full biofilm formation proficiency^23^. Therefore, we fed *C. elegans* on a mix of spores of the three biofilm-deficient mutant strains and measured the lifespan effect produced by this mixture. As shown in Fig. 3d, *C. elegans* fed on a mixture of the three biofilm-deficient mutant strains restored its lifespan to levels comparable with the lifespan obtained when the worm was fed on wild-type NCIB3610 cells. In this case, the worm lifespan fed on a mixture of the three biofilm mutant strains (Δ*bslA*+Δ*eps*+Δ*tasA*) increased by an average of 52.13% (average value, 29.59 days; 95% CI: 27.52–31.65; equality, *P*=0.003 by log-rank test) compared with the strains OP50 (average value, 19.45 days; 95% CI: 17.62–21.28) and NCIB3610 (average value, 31.28 days; 95% CI: 27.52–31.65; equality, *P*=0.002 by log-rank test) used as a food source (Supplementary Table 5). This lifespan effect correlates with the ability of the mixture of the three biofilm-defective strains (Δ*bslA*+Δ*eps*+Δ*tasA*) to recover full biofilm formation proficiency (see insert in Fig. 3d) and worm gut colonization (Fig. 3e). Neither JH642 cells that are poor biofilm makers^24^,^31^,^32^ (Supplementary Fig. 1) nor the biofilm-defective NCIB3610 derivates (Δ*tasA*, Δ*bslA* or Δ*epsG*) affected the growth and postembryonic development of *C. elegans* (worms fed on each biofilm mutant strain required an average of 42.5**±**2.0 h to reach the late L4/young adult stage). We observed no variation in food preference and/or food availability between the isogenic derivates of NCIB3610 cells as the reasons for the observed differences in the prolongevity effect of *B. subtilis* on *C. elegans*. Thus, the overall results indicated that biofilm formation proficiency constitutes a key positive property to fully understand the beneficial effect of *B. subtilis* on *C. elegans* lifespan (Table 1B).

### The biofilm and nitric oxide produce synergic anti-aging effects

**Figure 4:**
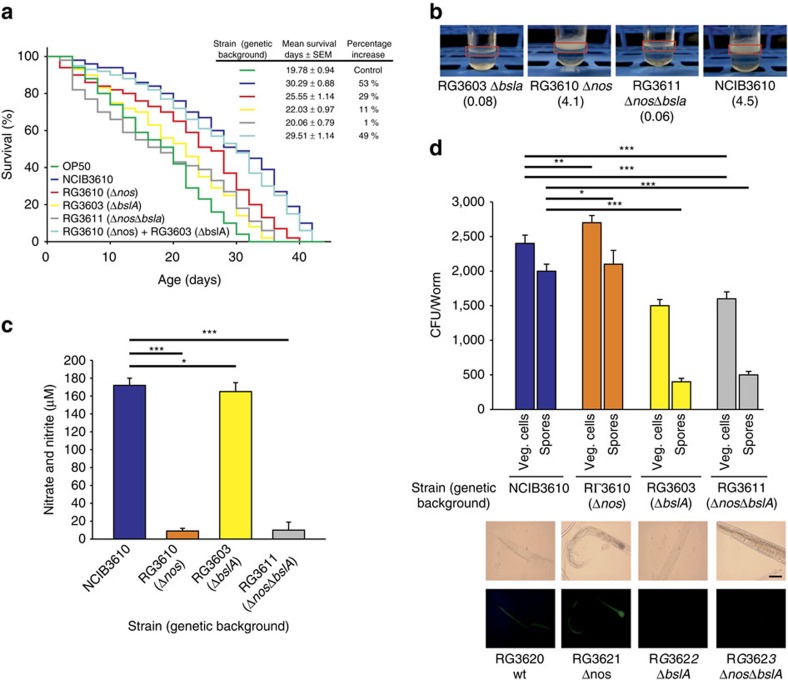
Biofilm formation and nitric oxide production are required to extend *C. elegans* lifespan. (**a**) Effects upon longevity of worms fed on undomesticated *B. subtilis* defective in biofilm production and/or NO synthesis. Fifty L4 worms were fed either on spores of undomesticated NCIB3610 (blue) or RG3610 (Δ*nos*, defective in NO production, orange) or RG3603 (Δ*bslA*, defective in biofilm synthesis, yellow) or RG3611 (Δ*nos*Δ*bslA*, defective both in NO production and biofilm synthesis, grey) or a mixture of RG3610 and RG3603, light blue) *B. subtilis* on NGM agar plates, and survival was monitored. A representative experiment**±**s.e.m. is shown. (**b**) Biofilm formation proficiencies of undomesticated *B. subtilis* strains harbouring mutations in genes essential for biofilm formation and/or NO production. Biofilms were developed in liquid NGM. (**c**) NO quantification of undomesticated *B. subtilis* strains harbouring mutations in genes essential for biofilm formation and/or NO production. The nitrite and nitrate concentrations are expressed as μM. Error bars show the mean**±**s.e.m. (*n*=3). ***P<0.001 and *P<0.05 (analysis of variance (ANOVA) with Bonferroni test). (**d**) Colonization of the worm gut by *B. subtilis* strains deficient in biofilm formation and/or NO production. L4 worms were allowed to develop on NGM agar plates seeded with OP50 cells, washed and transferred to fresh NGM plates containing spores of the wild-type NCIB3610 (blue) or RG3610 (Δ*nos*, defective in NO production, orange) or RG3603 (Δ*bslA*, defective in biofilm synthesis, yellow) or RG3611 (Δ*nos*Δ*bslA*, defective both in NO production and biofilm synthesis, grey) *B. subtilis* strains, respectively. The worms were taken and processed to measure the number of *B. subtilis* cells in the worm gut, expressed as CFUs per worm. Error bars show the mean**±**s.e.m. (*n*=3). ***P<0.001, **P<0.01 and *P<0.05 (ANOVA with Bonferroni test). Images shown on the bottom are epifluorescence micrographs of typically stained N2 *C. elegans* fed *epsA::gfp-*expressing *B. subtilis* strains (RG3620, wild-type; RG3621, Δ*nos*; RG3622, Δ*bslA*; and RG3623, Δ*nos*Δ*bslA*). The fluorescence images were superimposed to differential interference contrast (DIC) images to depict the localization of the labels within the cells. Scale bar, 20 μm.

The beneficial effect of domesticated *B. subtilis* over OP50 on *C. elegans* longevity has been recently attributed to the bacterium's nitric oxide (NO) production proficiency^28^. NO-mediated signalling plays key roles in all living organisms, and for an unknown reason, *C. elegans* is unable to produce its own NO but is able to incorporate the NO produced by *B. subtilis*^14^,^15^,^28^,^29^. Most organisms produce NO through aerobic conversion of L-arginine to L-citrulline in a reaction catalysed by the enzyme NO synthetaze encoded by the *nos* gene^55^. *E. coli* strains, several of which are routinely used to feed the worms (that is, OP50, HB101; refs 14, 15, 29), are not proficient in aerobic NO production because they lack a functional copy of *nos*^55^. However, *E. coli* can produce NO under anaerobic/microaerophilic conditions by a series of biochemical reactions associated with the anaerobic respiratory chain of the bacterium (Supplementary Table 6 and ref. 56). Under this scenario, *E. coli* might find permissive conditions for NO production in the oxygen-depleted environment of the worm intestine; therefore, the positive effect of *B. subtilis* on worm longevity^28^ could be due to at least one as-yet unknown additional factor apart from the intestinal NO provision. First, we were interested in determining whether the biofilm proficiency-dependent lifespan effect of *B. subtilis* on *C. elegans* was due to NO production^28^. As shown in Fig. 4a, the mutations affecting biofilm formation (Δ*bslA* mutant) or NO synthesis (Δ*nos* mutant) (Supplementary Table 1) produced a negative effect on *C. elegans* lifespan compared with the lifespan effect produced by the wild-type NCIB3610 strain. Interestingly, the negative effect on worm longevity was more pronounced when *C. elegans* was fed *B. subtilis* cells deficient in biofilm formation (but proficient in NO production; average value, 22.03 days; 95% CI: 20.13–23.94; equality, *P*=0.04 by log-rank test) compared with *B. subtilis* cells deficient in NO production (but proficient in biofilm formation; average value, 25.55 days; 95% CI: 23.31–27.79; equality, *P*=2.5 × 10^−7^ by log-rank test; Supplementary Table 7). The average worm lifespan increases of 29.17 and 11.38% were observed when the worms were fed on *B. subtilis* cells defective in NO or biofilm production, respectively, in comparison with the 53.13% lifespan increase (related to OP50 cells) obtained when the worms were fed on wild-type *B. subtilis* (average value, 30.29 days; 95% CI: 28.26–32.33; equality, *P*=0.00 by log-rank; Fig. 4a and Supplementary Table 7). Furthermore, the double mutant of *B. subtilis* deficient in biofilm formation and NO synthesis (Δ*nos-*Δ*bslA*; Supplementary Table 1) produced the most negative impact on worm survival (average value, 20.06 days; 95% CI: 18.52–21.60; equality, *P*=0.67 by log-rank test) with a final increase in worm survival of only 1.42% compared with OP50 cells (Fig. 4a and Supplementary Table 7). In addition, spore robustness and worm chemotactic behaviour toward Δ*nos* (60.0**±**2.0% of Δ*nos* spore survival after 30 s of treatment at pH=2.0 and chemotaxis index of 0.95**±**0.2, respectively) and Δ*nos-*Δ*bslA* (59.4**±**1.9% of Δ*nos-*Δ*bslA* spore survival after 30 s of treatment at pH=2.0 and chemotaxis index of 0.91**±**0.3, respectively) cells were not significantly different compared with the NCIB3610 strain. The different effects on *C. elegans* lifespan correlated with the capacity of each *B. subtilis* strain to form a biofilm (Fig. 4b), produce NO (Fig. 4c) and colonize the worm intestine (Fig. 4d). Thus, the positive lifespan effects of NO production and biofilm formation proficiency are different and synergistic.

If NO production and biofilm formation are distinct but complementary processes improving the longevity of *C. elegans*, then we hypothesize that a mixture of mutant *B. subtilis* cells, that is, cells deficient in biofilm formation (Δ*bslA*) and cells deficient in NO synthesis (Δ*nos*), would complement each other and restore the full lifespan effect of wild-type NCIB3610 cells on *C. elegans*. As predicted by this hypothesis, a mixture of Δ*bslA* and Δ*nos* cells (strains RG3610 plus RG3603, light blue curve in Fig. 4a) restored the worm lifespan to levels comparable to the effect of wild-type NCIB3610 cells (average value, 29.51 days; 95% CI: 27.77–31.75; equality, *P*=0.0141 by log-rank test) compared with OP50 cells (final increase in worm survival, 49.19%; Fig. 4a and Supplementary Table 7).

Overall, these results demonstrate that biofilm formation proficiency constitutes a new attribute, different from NO production, for the anti-aging effect of *B. subtilis* on *C. elegans* (Table 1B). The discovery that NO production is not the only factor that explains how *B. subtilis* extends the longevity of *C. elegans* opens the possibility of the existence of other factors that positively contributes to increase worm longevity.

### *B. subtilis* quorum sensing extends worm longevity

**Figure 5:**
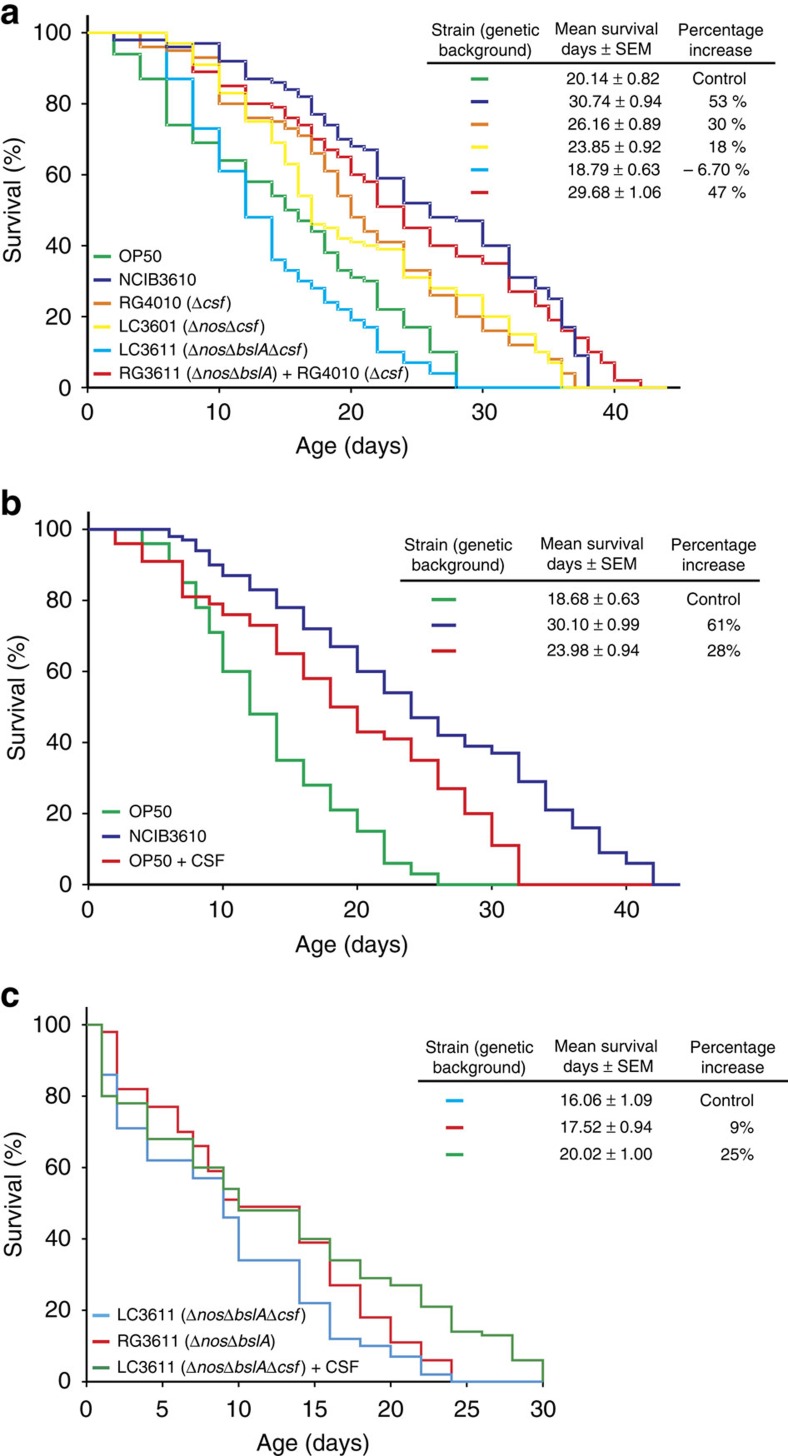
The quorum-sensing factor of *B. subtilis* (CSF) extends *C. elegans* longevity. (**a**) Synergic effects of biofilm formation proficiency (Δ*bslA*), NO production (Δ*nos*) and CSF synthesis (Δ*csf*) on *C. elegans* longevity. Late L4/young-adult-stage N2 worms were fed either on spores of undomesticated NCIB3610 (blue) or RG4010 (Δ*csf*, defective in CSF production, orange) or LC3601 (Δ*nosΔcsf*, defective in NO and CSF production, yellow) or LC3611 (Δ*nos*Δ*bslAΔcsf*, defective in NO production, biofilm synthesis and CSF production, light blue) or a mixture of RG3611and RG4010 (red) *B. subtilis* on NGM agar plates, and survival was monitored for signs of life. The data shown are the average**±**s.e.m. (*n*=3). (**b**) Exogenous CSF peptide increased the longevity of worms fed OP50 *E. coli*. Late L4/young-adult-stage N2 worms were fed either on OP50 *E. coli* (green) or OP50 *E. coli* plus 100 nM CSF peptide (red) on NGM agar plates, and the survival was monitored for signs of life. The data shown are the average**±**s.e.m. (*n*=3), and the survival increase is expressed as a percentage of the total number of worms fed OP50 *E. coli*. (**c**) Exogenous CSF peptide restored the longevity of worms fed *B. subtilis* deficient in QS. Late L4/young-adult-stage N2 worms were fed either on spores RG3611 (Δ*nos*Δ*bslA*, defective both in NO production and biofilm synthesis, red) or LC3611 (Δ*nos*Δ*bslAΔcsf*, defective in NO production, biofilm synthesis and CSF production, light blue) or LC3611 plus 100 nM CSF peptide (green) *B. subtilis* on NGM agar plates, and survival was monitored for signs of life. A representative experiment**±**s.e.m., and the survival increase as a percentage of the total number of worms fed on LC3611 cells is shown.

Intra- and interspecific quorum sensing (QS) constitutes a widely used strategy that bacteria use in nature to communicate with each other and with cells of different kingdoms^18^,^19^,^57^,^58^. Recent studies have shown the potential effect of bacterial QS on *C. elegans* physiology. *C. elegans* recognized *Vibrio cholerae* QS signalling molecules through the AWC^on^ neuron^59^. Furthermore, infection of *C. elegans* with *Candida albicans* or *Pseudomonas aeruginosa* was repressed by bacterial QS molecules^48^,^60^, increasing the idea that the nematode can detect microbial signals and thus modulate each other (bacteria and host) in a symbiotic relationship^61^. *B. subtilis* QS pentapeptide CSF (Competence Sporulation stimulating Factor, also named PhrC)^62^ was previously reported to contribute to intestinal homeostasis by activating key survival pathways of the host (p38 MAP kinase and protein kinase B) and by inducing cytoprotective heat shock proteins (Hsps)^21^,^63^. These effects of CSF^21^ depend on its uptake by the protein OCTN2, a host cell membrane transporter of organic cations present in the apical face of epithelial cells^63^. Because an orthologue of *octn2* is present in *C. elegans*^64^, we were interested in determining whether the CSF molecule affects the lifespan of *C. elegans*. As shown in Fig. 5, *C. elegans* fed on NCIB3610-isogenic *B. subtilis* strain deficient in CSF synthesis (Δ*csf* mutant, strain RG4010; Supplementary Table 1) showed a decreased lifespan compared with worms fed on wild-type NCIB3610 cells. In this case, worms fed on Δ*csf* cells showed an increase in survival of 29.89% (average value, 26.16 days; 95% CI: 24.41–27.91 days; equality, *P*=0.04 by log-rank test), compared with OP50 cells (average value, 20.14 days; 95% CI: 18.53–21.75; Fig. 5a and Supplementary Table 8).

When the longevity of worms fed on the single, double and triple isogenic mutant strains RG4010 (Δ*csf*), LC3601 (Δ*nos*Δ*csf*) and LC3611 (Δ*nos*Δ*csf*Δ*bslA*) (Supplementary Table 1) was compared, a synergic decrease in the worm lifespan was observed (Fig. 5a). The average survival of worms fed RG4010 (Δ*csf*), LC3601 (Δ*csf*Δ*nos*) or LC3611 (Δ*csf*Δ*nos*Δ*bslA*) cells was 26.16 days (95% CI: 24.41–27.91 days; equality, *P*=0.04 by log-rank test), 23.85 days (95% CI: 22.05–25.65 days; equality, *P*=2.5 × 10^−6^ by log-rank test) and 18.79 days (95% CI: 17.55–20.03 days; equality, *P*=0.7695 by log-rank test), respectively, compared with OP50 cells (Fig. 5a and Supplementary Table 8).

Interestingly, the *B. subtilis* triple mutant strain (deficient in biofilm formation and NO and CSF production, strain LC3611) produced a lifespan effect on *C. elegans* survival that was smaller than the effect obtained with OP50 cells used as a control (6.7% lifespan decrease, Fig. 5a). In addition, the lifespan effects of the single (Δ*cfs*), double (Δ*csf*Δ*nos*, *ΔbslAΔnos*) and triple (Δ*bslA*Δ*nos*Δ*csf*) mutant strains (Fig. 5a and Supplementary Fig. 5) correlated well with the proficiency or deficiency in biofilm formation and worm gut colonization of each strain, and there was no significant difference in spore robustness or worm chemotactic behaviour toward these mutant cells compared with the NCIB3610 strain (Supplementary Fig. 1).

The use of a mixture of Δ*bslA*Δ*nos* cells (deficient in biofilm formation and NO synthesis) and Δ*cfs* cells (deficient in CSF synthesis) to feed the worm (average worm survival obtained with each strain individually used as food: 20.06 days and 26.16 days, respectively; Supplementary Tables 7,8) complemented the deficiency of each mutant strain and re-established the lifespan of *C. elegans* to levels comparable to the lifespan effect produced by wild-type *B. subtilis* cells. Average worm survival values of 30.74 days (95% CI: 28.9–32.58 days; equality, *P*=0.003 by log-rank test) and 29.68 days (95% CI: 27.59–31.77 days; equality, *P*=0.004 by log-rank test) were obtained with wild-type cells and mixture of Δ*bslA*Δ*nos* plus Δ*cfs* cells, respectively (Fig. 5a and Supplementary Table 8). Accordingly, the use of FUdR did not interfere with the effects produced by the *ΔbslA*, *Δnos* and Δ*cfs B. subtilis* mutant strains on *C. elegans* survival (Supplementary Table 9).

To confirm that CSF production constitutes a novel prolongevity factor for *C. elegans*, we supplemented the worm diet with pure CSF (100 nM) in Petri dishes containing OP50 (Fig. 5b) or LC3611 (Δ*bslA*Δ*nos*Δ*csf*; Fig. 5c) bacterial cells as a worm food source. Interestingly, the added CSF increased the lifespan of *C. elegans* fed on OP50 cells, with average survival values of 18.68 days (95% CI: 17.44–19.92 days) and 23.98 days (95% CI: 22.14–25.85 days; equality, *P*=0.0001 by log-rank test) in the absence or presence of CSF supplementation, respectively (Fig. 5b and Supplementary Table 8). Similarly, the addition of CSF to *C. elegans* fed on the *B. subtilis* mutant strain LC3611 (Δ*bslA*Δ*nos*Δ*csf*) increased the worm lifespan to levels even higher than those obtained when Δ*bslA*Δ*nos* cells (strain RG3611, proficient in CSF production) were used as a food source (Fig. 5c). Average worm survival values of 16.06 days (95% CI: 13.93–18.19 days), 17.52 days (95% CI: 15.55–19.49 days; equality, *P*=0.15 by log-rank test) and 20.02 days (95% CI: 18.48–21.56 days; equality, *P*=0.55 by log-rank test) were obtained with the Δ*bslA*Δ*nos*Δ*cfs*, Δ*bslA*Δ*nos* and Δ*bslA*Δ*nos*Δ*cfs* (plus CSF) cells, respectively (Fig. 5c and Supplementary Table 8). In sum, these results confirm the QS molecule CSF as the third factor that contributes to the lifespan extension effect of *B. subtilis* on *C. elegans* (Table 1B). In addition, these results strongly suggested that the positive lifespan effect of *B. subtilis* on *C. elegans* is primarily, or exclusively, dependent on the three properties studied here (that is, NO, CSF and biofilm production), which produced positive synergic effects on worm survival.

Using Cox proportional hazard model (Supplementary Table 10), we confirmed that the biofilm formation proficiency (absence or presence of *bslA* activity) constitutes the more important *B. subtilis* property responsible for extending worm longevity. Worms fed on biofilm-deficient *B. subtilis* have a risk of death 2.45-fold higher than worms fed on biofilm-proficient cells (Supplementary Table 10). Interestingly, this statistical analysis showed that the effects of NO and CSF on lifespan were not additive and independent one from the other but showed the existence of a genetic dependence between *nos* and *csf*. Up to now, we do not know the molecular reason for this bacterial gene interaction in the worm gut environment but it shows up the complexity of the mechanism responsible for the extended host longevity. We are currently investigating this interesting finding.

### The *B. subtilis* biofilm enhances the production of NO and CSF

The cell-to-cell communication and division of labour that occurs inside the biofilm produce significant differences in gene expression compared with the gene expression pattern of cells grew under planktonic conditions^16^,^19^,^23^,^58^. In this sense, the levels of NO and CSF produced in *B. subtilis* cultures have been measured only under planktonic growth conditions^62^. To determine whether NO and/or CSF production is affected in *B. subtilis* cells that develop as a biofilm, we grew NCIB3610 cells under planktonic and biofilm supporting conditions and measured the production levels of the two prolongevity molecules. As shown, the production levels of NO (Table 2) and CSF (Table 2) were significantly enhanced when *B. subtilis* was grown as a biofilm compared with the levels of NO and CSF produced under planktonic conditions. These results (Table 2 and Figs 2 and 3) reinforce the importance of the biofilm formation proficiency in the longevity of *C. elegans*.

The results shown in Table 2 also suggest that *B. subtilis* viability inside the worm gut would be an important prerequisite for the prolongevity effect. Only alive (and not dead) vegetative *B. subtilis* cells, originated from worm gut germinated spores, are able to form a biofilm and enhance production of the anti-aging molecules NO and CSF. If this interpretation is correct, there should be a difference in the magnitude of the prolongevity effect between worms fed on alive or dead *B. subtilis* cells. To test this hypothesis, we fed *C. elegans* on dead and alive *B. subtilis* spores to measure the magnitude of the produced lifespan. Interestingly, *B. subtilis* must be alive in order to produce its beneficial prolongevity effect (Supplementary Fig. 6 and Supplementary Table 11). Dead *B. subtilis* used to feed *C. elegans* produced a lower (−14.32.0%) lifespan effect (average survival, 25.79 days; 95% CI: 24.22–27.36 days; equality, *P*=0.000002 by log-rank test) than alive *B. subtilis* (average survival, 30.10 days; 95% CI: 25.35–29.29 days) (Supplementary Fig. 6 and Supplementary Table 11).

Accordingly, dead pathogenic bacteria (that is, killed *P. aeruginosa*) used to feed *C. elegans* increase worm survival in comparison with live pathogenic bacteria used as a food source. In addition, *E. coli* strains, including the ‘non-virulent' OP50 strain, are mildly pathogenic to aging *C. elegans*^14^,^15^,^29^. We found that dead OP50 cells effectively prolonged *C. elegans* survival (21% lifespan increase), with average survival of 24.73 days (95% CI: 23.23–26.23 days; equality, *P*=2.4 × 10^−7^ by log-rank test) in comparison with live OP50 cells (average survival, 20.51 days; 95% CI: 19.35–21.67 days) used as food sources. Overall, these results confirmed that *B. subtilis* constitutes a beneficial flora for *C. elegans* and showed that the prolongevity effect of this bacterium depends on its viability inside the worm gut.

### *B. subtilis* downregulates the insulin-like signalling pathway

The lifespan of *C. elegans* is subject to regulation by conserved signalling pathways and transcription factors that sense stress, environmental cues and nutrient availability^29^. Dietary restriction (DR) and the insulin-like signalling (ILS) pathway are central for the regulation of longevity in different animal models, including *C. elegans*^1^,^2^,^65^,^66^,^67^,^68^. These longevity regulatory pathways (DR and ILS) converge on the positive and negative regulation of the transcription factors DAF-16 and HSF-1, repectively^68^,^69^,^70^.

**Figure 6:**
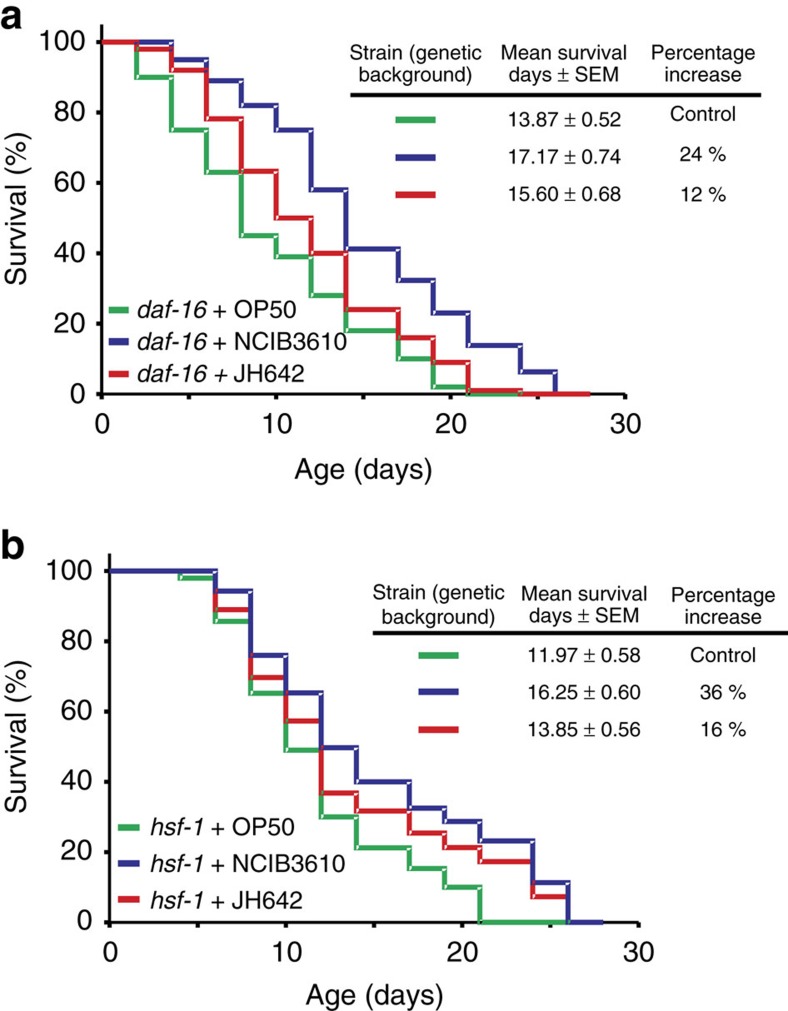
The prolongevity effects of *B. subtilis* require the activity of the anti-aging factors DAF-16 and HSF-1. Undomesticated and domesticated *B. subtilis* cells (NCIB3610 and JH642, respectively) partially extend the lifespan of *daf-16* and *hsf-1* worms compared with their lifespan effect produced on N2 worms. Late L4/young-adult-stage *daf-16* (**a**) or *hsf-1* (**b**) worms were fed either on spores of undomesticated NCIB3610 (blue) or domesticated JH642 (red) *B. subtilis* on NGM agar plates, and the survival was monitored for signs of life. Each graph is the average**±**s.e.m. (*n*=3), and the survival increase is expressed as a percentage of the total number of worms fed on OP50 *E. coli* (green).

To determine how *B. subtilis* affects the signalling pathways involved in the regulation of *C. elegans* longevity, we fed *daf-16* (mu86) and *hsf-1* (sy441) mutant worms (strains CF1038 and PS3551, respectively, Supplementary Table 1) with wild-type *B. subtilis* and isogenic mutants showing a decreased effect on wild-type N2 worm lifespan (Δ*bslA,* Δ*nos* and Δ*csf* cells). Interestingly, the lifespan extension effect of wild-type *B. subtilis* on *daf-16* and *hsf-1* worms was approximately half of the lifespan extension effect on wild-type N2 worms (Fig. 6a,b and Supplementary Table 12). While the average lifespan extension effect of NCIB3610 and JH642 cells (compared with OP50 cells) on wild-type (N2) worms was 55.54 and 27.13%, respectively (Table 1A), in *daf-16* worms, these percentages were 23.79 and 12.47%, respectively (Fig. 6a and Supplementary Table 12). Similarly, the lifespan extension effect on *hsf-1* worms fed on NCIB3610 or JH642 cells (Fig. 6b) was partial in comparison with the lifespan extension effect on wild-type worms fed the same type of *B. subtilis* cells. In this case, the average lifespan extension effect produced by NCIB3610 and JH642 cells (compared with OP50 cells) on *hsf-1* worms was 35.76% and 15.71%, respectively (Fig. 6b and Supplementary Table 12). Because HSF-1 and DAF-16 are active in *daf-16* and *hsf-1* worms, respectively^69^, our results suggest that the prolongevity effect of *B. subtilis* on *C. elegans* depends on the activity of DAF-16 and HSF-1 (Table 1C,D).

**Figure 7:**
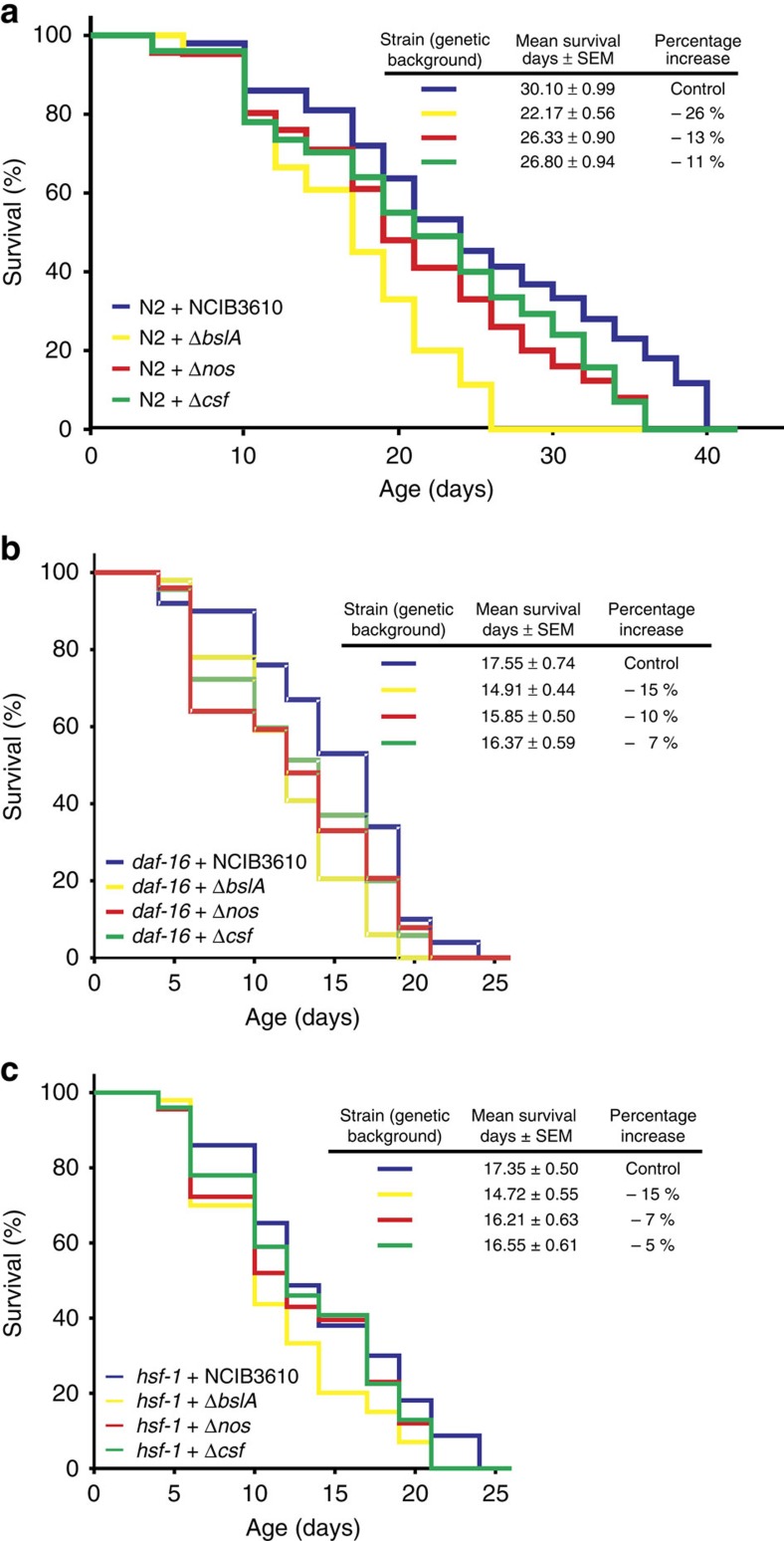
Dissecting the contribution of each *B. subtilis* anti-aging property on worm survival pathways. Late L4/young-adult-stage N2 (**a**), *daf-16* (**b**) or *hsf-1* (**c**) worms were fed either on spores of RG3610 (Δ*nos*, defective in NO production, red) or RG3603 (Δ*bslA*, defective in biofilm synthesis, yellow) or RG4010 (Δ*csf*, defective in CSF production, green) *B. subtilis* on NGM agar plates, and the survival was monitored for signs of life. Undomesticated NCIB3610 *B. subtilis* (blue) was used as a control. A representative experiment**±**s.e.m. (*n*=3) is shown, and the survival increase is expressed as a percentage of the total number of worms fed on NCIB3610 cells (blue).

How does each *B. subtilis* prolongevity property depend on DAF-16 and/or HSF-1 activities? To answer this key question, we measured the survival of N2, *daf-16* and *hsf-1* worms fed on NCIB3610-isogenic mutants defective in biofilm (Δ*bslA*), NO (Δ*nos*) and CSF (Δ*csf*) production (Fig. 7). The abolishment of each of the *B. subtilis* prolongevity properties was responsible for partial lifespan decreases of 26.35, 12.52 and 10.96% for wild-type N2 worms fed on Δ*bslA-*, Δ*nos-* and Δ*csf- B. subtilis* cells, respectively, compared with the use of wild-type NCIB3610 cells (Fig. 7a and Supplementary Table 13). Accordingly, the survival of *daf-16* and *hsf-1* worms fed on biofilm-deficient (Δ*bslA*) *B. subtilis* cells showed a lifespan decrease of 15.04% (average value, 14.91 days; 95% CI: 14.04–15.78 days) and 15.16% (average value, 14.72 days; 95% CI: 13.65–15.79 days), respectively, compared with the average lifespan obtained with wild-type NCIB3610 cells used to fed *daf-16* (average value, 17.55 days; 95% CI: 16.48–18.62 days) and *hsf-1* worms (average value, 17.35 days; 95% CI: 16.18–18.52 days) (Fig. 7b,c and Supplementary Table 13). Similarly, the average worm survival percentage decreased by 9.69% (average value, 15.85 days; 95% CI: 14.67–17.03 days) and 6.57% (average value, 16.21 days; 95% CI: 14.98–17.44 days) in *daf-16* and *hsf-1* worms, respectively, when fed on *B. subtilis* cells defective in NO synthesis (Δ*nos*) in comparison with feeding *daf-16* and *hsf-1* worms wild-type NCIB3610 cells (Fig. 7b,c and Supplementary Table 13). Finally, survival decreased by 6.72% (average value, 16.37 days; 95% CI: 15.20-17.54 days) and 4.61% (average value, 16.55 days; 95% CI: 15.35–17.75 days) in *daf-16* and *hsf-1-* worms, respectively, when fed on Δ*csf B. subtilis* cells compared with feeding wild-type NCIB3610 cells to *daf-16* and *hsf-1* worms (Fig. 7b,c and Supplementary Table 13). In addition, we confirmed that the lifespan effects produced by *B. subtilis* NCIB3610 and its isogenic derivates (Δ*nos*, Δ*bslA* and Δ*csf*) on wild-type *daf-16* and *hsf-1* worms were not affected by the inclusion of FUdR in the assays (Supplementary Table 9). In sum, these results indicated that each of the three anti-aging properties of *B. subtilis* require the activity of HSF-1 and DAF-16 (ref. 69; Fig. 7 and Supplementary Tables 12,13) and reinforced the former conclusion suggesting that the biofilm formation proficiency of *B. subtilis* is the most important property causing the beneficial effect on *C. elegans* longevity.

**Figure 8:**
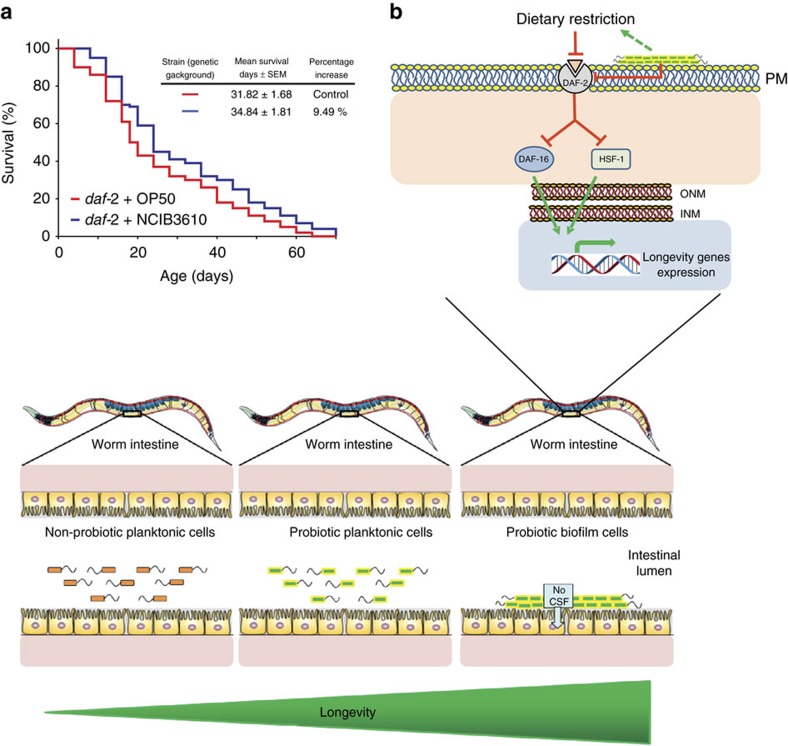
The prolongevity effect of *B. subtilis* is primarily transduced through the ILS pathway. (**a**) L4 *daf-2* worms were fed either NCIB3610 *B. subtilis* (blue) or OP50 *E. coli* (red) on NGM agar plates, and the survival was monitored for signs of life. A representative experiment**±**s.e.m. and the survival increase as a percentage of the total number of worms fed on OP50 *E. coli* cells is shown. (**b**) A workable model of how *B. subtilis* improves host health and longevity. Bottom illustration. Once *B. subtilis* forms a biofilm in the intestine of *C. elegans* (right cartoon), a continued and probably coordinated provision of beneficial and anti-aging NO and CSF molecules to the host tissues is produced. In addition, if NO and CSF are synthesized in the intestinal lumen, because the biofilm is not formed (left and middle cartoons), they would rapidly disappear because of their dilution before reaching the host tissues, the presence of degrading enzymes (that is, intestinal peptidases for CSF) or the intrinsic short-life of the molecule (that is, NO). Top illustration. The lifespan of the worm is regulated by a biochemical pathway that influences the activity of the gene-transcription factors DAF-16 and HSF-1 The binding of insulin-like molecules activates DAF-2, which in turn activates a series of protein kinase enzymes (not shown here for simplicity) that phosphorylates DAF-16, keeping it inactive in the cytoplasm (**⊥**). In addition, active DAF-2 is responsible for the formation of an inhibitory protein complex (not shown here for simplicity) that sequesters HSF-1 in the cytoplasm (**⊥**). On downregulation of DAF2, DAF-16 and HSF-1 become active after phosphorylation and trimerization, respectively (not showed here). Active DAF-16 and HSF-1 are translocated to the nucleus and activated stress-protective and -prolongevity genes (green arrow). The biofilm established by *B. subtilis* or a signal derived from it, produces a direct (solid line) or indirect (dashed line) downregulation (**⊥**) of DAF-2 receptor, activating the downstream cascade and extending lifespan (see text for details).

As mentioned, host longevity is mainly regulated by the signalling pathways of DR and the nutrient-sensing pathway ILS that converge in the regulation of DAF-16 and HSF-1 activities^68^,^69^,^70^. Interestingly, ILS could mediate part of the DR prolongevity effect because DR downregulates the activity of ILS^1^,^2^. Therefore, we tested the putative participation of the ILS pathway in the prolongevity effect of *B. subtilis* on *C. elegans*. DAF-2 is the *C. elegans* homologue of the insulin-like receptor (IGFR), a transmembrane receptor that negatively regulates DAF-16 and HSF-1 (ref. 69). Loss-of-function mutations affecting DAF-2 have been shown to increase longevity and resistance to heat stress^68^. Interestingly, most of the positive lifespan effects of *B. subtilis* on wild-type *C. elegans* disappeared when this bacterium was used to feed *daf-2* (e1370) worms. Average values of 31.82 days (95% CI: 28.84–35.10 days) and 34.84 days (95% CI: 31.26–38.39 days) were obtained when *daf-2* worms were fed on OP50 and NCIB3610 cells, respectively (Fig. 8a and Supplementary Table 14). These lifespan values represent a 9.49% increase in the longevity of NCIB3610-fed *daf-2* worms (Fig. 8a) versus a 55.54% average increase in the longevity of NCIB3610-fed N2-worms compared with OP50 cells used to feed *daf-2* and N2 worms, respectively (Table 1A,E). These results strongly indicated that the anti-aging effect of *B. subtilis* on *C. elegans* is mainly transduced through the ILS pathway.

Aging is a multifactorial and poorly understood process characterized by progressive impairment of the host response to stresses and general cellular deterioration of key metabolic pathways^1^,^2^. Intensive work in different animal models (that is, yeasts, worms, flies, mice and monkeys) has identified a number of factors that promote longevity. Restricting food intake (DR), decreasing insulin/IGF-1 signalling (IIL), slowing mitochondrial respiration, reducing germline function or lowering temperature can all extend lifespan^1^,^2^. In addition, probiotics are associated with a broad spectrum of positive effects on host health, including positive effects on host longevity^9^,^10^,^11^,^12^,^13^,^14^,^15^,^21^,^22^.

To our knowledge, the present study is the first to formulate and address the question of how the bacterial biofilm might affect the prolongevity effect of beneficial gut bacteria on the host. Our evidence directly implicates the biofilm formation proficiency as the primary cause of the increased lifespan and stress resistance (that is, healthy longevity) of *C. elegans* when fed *B. subtilis* (Figs 1, 2, 3 and Table 1).

In the case of the host (*C. elegans*), the benefits of the biofilm are evident: lifespan extension, higher stress resistance and probably competitive exclusion of pathogens^2^,^21^,^22^,^42^. In addition to our results, it has been reported that *B. subtilis* strains proficient in biosurfactan production (that is, fengycin) are able to protect *C. elegans* from infection by bacterial pathogens^42^. In addition, the ability to establish a biofilm in connection with the host cells would allow for a stronger and closer interaction between the bacterium and the host^9^,^44^. The finding that exogenously added CSF (Fig. 5) and NO^28^ extend the lifespan of *C. elegans* fed *E. coli* cells supports the notion of the underlaying phenomenon of worm–bacteria QS. This dual microbial–worm interaction would allow the bacterium to colonize and establish a multicellular biofilm in the friendly environment of the worm gut mucosa, a similar scenario to the formation of stable biofilms during the beneficial bacteria–plant interaction of some Bacilli (that is, *B. subtilis* and *B*. *amyloliquefaciens*) with PGPR (Plant Growth Promoting Rhizobacteria) activity on plant roots (rhizospheric biofilm)^23^. In addition, the bacterial properties and behaviour in a multicellular biofilm differ from the behaviour and properties of bacteria grown as individuals under planktonic conditions^16^,^17^,^18^. Specifically, our results demonstrated the importance of the biofilm for the success of *B. subtilis* to healthy *C. elegans* gut colonization (Figs 2 and 3), increase the production of the anti-aging molecules NO/CSF (Table 2), and increase worm longevity (Figs 1, 4 and 5). Future studies would address how the production of the anti-aging molecules NO and CSF is enhanced in *B. subtilis* cells grown as a biofilm (Table 2) and whether this higher NO and CSF production might be influenced by signals originating from the host (Supplementary Table 10).

The present work also indicated that the prolongevity effect of *B. subtilis* on *C. elegans* requires the activity of FOXO/DAF-16 and HSF-1 (Figs 6 and 7 and Table 1). At the top of the regulatory pathways that modulate the activity of HSF-1 and DAF-16 in *C. elegans* are the ILS pathway (homologous to the insulin/IGF-1 pathway present in humans) and the process of DR^1^,^2^,^29^. We showed that the prolongevity effect of *B. subtilis* on *C. elegans* primarily depends on the functionality (downregulation) of the ILS pathway (Fig. 8a). This result points to the ILS pathway as the target of the *B. subtilis* biofilm to increase host longevity. Interestingly, it has been observed that healthy human centenarians likely have IGF-1 receptor genetic variants associated with reduced function, an intriguing observation that correlates with our findings. As ILS pathway is part of the nutrient signalling pathway, it is also (negatively) controlled by DR a scenario that open the possibility that ultimately DR might sense and control the ILS-dependent prolongevity effect of *B. subtilis* (Fig. 8b). Finally, driven by the Microbiome Project^3^,^8^,^10^,^71^, literature about the microbiota and its effects on human health and longevity has grown exponentially in the last decade. This scenario and the present results open the possibility to test if *B. subtilis* might produce beneficial effects on human longevity^72^,^73^,^74^.

## Methods

### Strains and growth media

The *B. subtilis* strains used in this study were the domesticated JH642 (ref. 33), the undomesticated RG4365 (ref. 27) and NCIB3610 (ref. 23), and their isogenic derivatives (Supplementary Table 1). The OP50 *E. coli* strain was obtained from the *Caenorhabditis* Genetic Center (CGC). As indicated, bacteria were grown in Luria-Bertani (LB) broth and Schaeffer's sporulation medium (SM)^30^. Sporulation was induced by the starvation method: *B. subtilis* strains were grown in SM medium at 37 **°**C for 48 h (ref. 30). After this incubation period, the culture was heat-treated for 20 min at 80 °C to kill vegetative cells^30^. To obtain pure spores, the heat-treated culture was treated three times with lysozyme (25 μg ml^−1^; Sigma Co.), washed each time with cold water and centrifuged until 100% of the culture consisted of phase-bright spores. When indicated, the pure spore solution was autoclaved twice to kill spores. Wild-type N2 (Bristol) and mutant strains (obtained from CGC) worms were maintained at 20 °C on Nematode Growth Medium agar (NGM)^28^ plates seeded with OP50 *E. coli* or *B. subtilis* cells with or without supplementation with ampicillin (100 μg ml^−1^), respectively. If necessary, the antifungal amphotericin (25 μg ml^−1^; Sigma Co.) was also added to the NGM medium. The QS pentapeptide (ERGMT) was obtained from Thermo Scientific Co. (formerly Invitrogen Co.) and conserved at −20 °C until use.

### Lifespan analysis

Lifespans were monitored at 20 °C as described previously^28^. Briefly, to obtain a synchronized population, embryos were isolated by treating adult worm hermaphrodites with alkaline hypochlorite and allowing them to develop. In all the cases, late L4/yound adult stage worms were used at t=0 for lifespan analysis, and 16 μM 5-fluoro-2′-deoxyuridine (FUdR, Sigma Co.) was used to inhibit progeny growth. During the quantification of the number of dead/live worms, the investigator was blinded to the group allocation. All the experiments were repeated at least three times. Worms were considered dead when they ceased pharyngeal pumping and did not respond to prodding with a platinum wire. Worms with internal hatching were removed from the plates and excluded from lifespan calculations. Given that some temperature-sensitive mutations in some genes lead to the formation of dauer larvae, the lifespan experiments involving *daf-2*(e1370) were performed shifting the temperature from 15 to 20 °C, the non-permissive temperature, at late L4/young adult stage^1^. For lifespan analysis with no FUdR, a synchronous population was obtained by sodium hypochlorite treatment of gravid hermaphrodites to obtain eggs that were then raised on standard NGM plates. Late L4/young adult stage worm were transferred to fresh plates in the complete absence of FUdR every 2 days, while any of them were fertile. Survival was scored as described above.

### Lifespan after different stress assays

Thermotolerance assays were performed as described^28^. The worms were allowed to develop and grow on NGM agar plates at 20 °C until they reached late L4/young adult stage. Then, the worms were shifted to 34 °C as 5-day-old adults. In a typical experiment, NGM plates containing ∼50 worms per plate were used. Single plates were removed (by triplicate) from the high temperature at the time intervals shown in the figures, and the worms were scored for signs of life as described in the ‘Lifespan Analysis' above. Osmotic, metal and oxidative stress assays were performed as described. The worms were allowed to develop and grow on NGM agar plates at 20 °C until they reached late L4/young adult stage. Then, we transferred ∼70 worms as 5-day-old adults from agar plates to each 24-well plate containing 200 mM NaCl (for osmotic stress), 50 μM cadmium (for metal stress), 25 mM H_2_O_2_ (for oxidative stress) and incubated the plates at 20 °C. At different time intervals, the worms were scored for signs of life as described in the ‘Lifespan Analysis' above. To calculate the median survival time, each data set was fitted to a Boltzmann sigmoid curve; the average**±**s.e.m. is presented in the figures.

### Culturing bacteria from worms

The N2 *C. elegans* eggs were isolated using a solution of 10% commercial bleach and 1 N NaOH followed by four washes with M9 buffer (22 mM KH_2_PO_4_, 42 mM Na_2_HPO_4_, 85 mM NaCl and 1 M MgSO_4_). Approximately 500 eggs were transferred to a 60 mm plate with NGM agar and incubated overnight at 20 °C with agitation to allow L1 larvae to emerge. Then, approximately 500 L1 larvae per experiment were grown for 48 h on NGM plates (a time that allows worm development to reach the L4 larvae stage) seeded with OP50 *E. coli* cells (1 × 10^5^ cells per plate) or spores (1 × 10^5^ spores per plate) of each *B. subtilis* strain. At different incubation times, 50 worms were transferred to Eppendorf tubes containing M9 buffer, and 1% Triton X-100. The worms were treated with 25 mM levamisole to induce temporal paralysis, superficially sterilized with 3% commercial bleach for 15 min and washed three times with M9 buffer. After the worms were surface sterilized, worms devoid of outside bacteria were disrupted using a pellet pestle (Sigma Co.), centrifuged and resuspended in 500 μl M9 buffer. Finally, 50 μl of each cell suspension was used to prepare serial dilutions of the bacteria before counting. To this end, 100 μl of the appropriate serial dilutions was spread with a Drigalski scraper on LB Petri dishes. The number of colony-forming units (CFUs) was determined after 24 h of incubation at 37 °C. To determine the number of spores, samples were heat-treated at 80 °C for 20 min before spreading. This treatment kills the vegetative cells, while the spore cells remain alive^33^. The number of CFUs obtained before and after the heat treatment represents an estimation of the number of vegetative and spore cells, respectively, inside the worm.

### β-Galactosidase assay

β-Galactosidase activity was assayed in *B. subtilis* cells harbouring *lacZ* fusions, and the specific activity was expressed in Miller Units (MU)^30^. To determine gut-derived β-galactosidase activity, 1-day-old L4 worms were separately fed on NGM plates with domesticated and undomesticated *B. subtilis* that harboured the p_*srf-lacZ*_ reporter fusion. At different times, approximately 100 worms were taken from the NGM plates containing each *B. subtilis* type, washed, disrupted and processed as indicated in ‘Culturing bacteria from worms' to measure the β-galactosidase expression level of each worm extract.

### Microscopic visualization of bacterial cells colonizing the nematode intestinal tract

Day 5 adult worms fed *B. subtilis* strains carrying an integrated *epsA::gfp* or *bslA::gfp* reporter fusion were pipetted out from plates using M9 buffer and 0.25 mM sodium azide (Sigma Co.) and covered with a glass coverslip. Bacterial colonization of the nematode digestive tract was observed using an Olympus FV1000 laser confocal scanning microscope. Images were analysed with Olympus software and Fiji software.

### Bacterial pulse-chase experiment

Day 5 adult worms fed different *B. subtilis* strains (JH642 or NCIB3610) were washed and transferred to NGM plates containing the indicated new bacterial cells (that is, OP50, JH642 and/or NCIB3610) at a titre of 1 × 10^6^ CFUs per plate as a new food source. At different times (indicated in the figure legends), the number of bacteria colonizing the worm intestine was calculated as described in the ‘Culturing bacteria from worms'.

### Determination of NO and CSF

*B. subtilis* strains were grown under planktonic or biofilm-supporting conditions (with or without shaking, respectively) in MSgg^31^ or NGM^28^ broth at 25 **°**C for 36 h. Then, the supernatants of each culture were taken and filter sterilized. Appropriate aliquots of each cell-free supernatant were assayed for the presence of NO-derived metabolites (NO_2_^−^ and NO_3_^−^) as reported previously^28^. For determining the β-galactosidase activity driven from the *csf*-promoter, the cells were grown as a biofilm or planktonically, concentrated by centrifugation and treated as reported previously^30^. The production levels of NO and CSF are expressed as μmol and MU, respectively, per 1 × 10^8^ CFUs counted from the cultures developed under planktonic and biofilm-supporting conditions.

### Self-brood size and egg production rate

These experiments were performed essentially as described^28^. The eggs from strain N2 nematodes were isolated, treated with hypochlorite and incubated for 20 h at 20 °C in S-buffer (0.5 M KH_2_PO_4_, 0.5 M K_2_HPO_4_ and 0.1 M NaCl). A synchronized population of L1 arrested worms was then placed on NGM agar plates seeded with OP50 *E. coli* or NCIB3610/JH642 *B. subtilis* cells. Five late L4/young adult stage animals were picked manually and transferred to a new plate. The worms were transferred twice a day to prevent overcrowding until egg laying ceased. The progeny was counted 3 days after removal of the parents. The experiment was performed in triplicate.

### Postembryonic development and adult size

These experiments were performed as described previously^28^. Strain N2 worms were grown on NGM agar plates seeded with OP50 *E. coli* or NCIB3610/JH642 *B. subtilis*. Unstaged eggs were placed at 20 °C and allowed to hatch for 4 h. Larvae that hatched during this period were placed singly on fresh plates and monitored every 5 h until they reached late L4/young adult stage. To measure the worm size, ∼20 randomly picked worms fed OP50 *E. coli* or NCIB3610/JH642 *B. subtilis* cells were anaesthetized with sodium azide, straightened and photographed. Their length in pixels was then compared with a 1 mm scale bar.

### Chemotaxis assay

Batches of 20 age-synchronized worms (late L4/young adult stage) were transferred to a plate containing lawns of OP50 *E. coli* or JH642 or NCIB3610 *B. subtilis* with a diameter of ∼0.5 mm. The number of worms on each bacterial lawn was counted after 12 h of incubation at 20 °C and the choice index was calculated. Chemotaxis of an individual animal was measured by watching the tracks that they left on the agar. The choice index was calculated as follows:

Chemotaxis index CI: No. of worms in tested bacteria−No. of worms on OP50/Total number of used worms. If CI=(−1.0) represents complete preference for OP50 *E. coli*, (+1.0) represents complete preference for the test bacterium and (0.0) represents an equal distribution.

### Statistical analysis

All assays were performed in at least duplicate or triplicate. Mean survival days, standard error, intervals of mean survival days with 95% confidence and equality *P* values to compare averages were calculated by log-rank and Kaplan–Meier tests using OASIS programme. Cox' regression model was used to estimate the relative risk of worm death under the different *B. subtilis* treatments compared with the control group: *h*_i_(t)=*h*_0_(t)exp(*β*_1_*x*_i1_+*β*_2_*x*_i2_+...+ *β*_k_*x*_ik_), where *h*_i_(t) and *h*_0_(t) denote the conditional hazard and baseline hazard rates, respectively; *β*_i_ is the unknown parameter for treatment group; and *x*_i_ takes values 0 and 1, being an indicator variable for two samples, the control and treatment group. Figures concerning the obtained quantitative information were prepared by Sigma Plot 10.0 or OriginPro 8.0 programmes.

### Data availability

The authors declare that all data supporting the findings of this study are available within the article and its Supplementary Information files, or are available from the corresponding author on reasonable request.

## Additional information

**How to cite this article:** Donato, V *et al. Bacillus subtilis* biofilm extends *Caenorhabditis elegans* longevity through downregulation of the insulin-like signalling pathway. *Nat. Commun.* **8**, 14332 doi: 10.1038/ncomms14332 (2017).

**Publisher's note:** Springer Nature remains neutral with regard to jurisdictional claims in published maps and institutional affiliations.

## Supplementary Material

Supplementary Information Supplementary Figures and Supplementary Tables.

## Figures and Tables

**Table 1:**
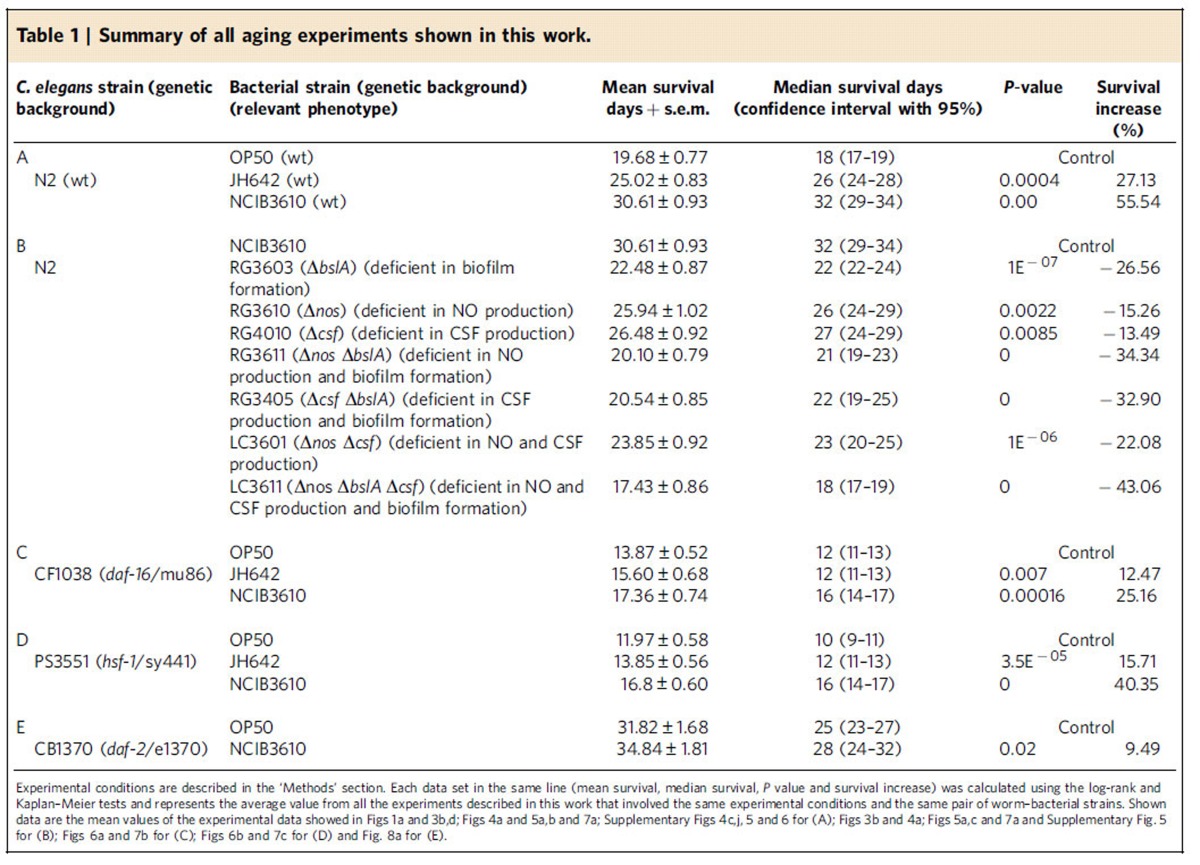
Summary of all aging experiments shown in this work.

*Reaction conditions:**1**/LiHMDS/**2**/[Pd(*η*^3^-C_3_H_5_)Cl]_2_/S-IPr·HCl=200/200/100/2.5/5; 0.1 M of ketone **1**; T=30^o^C; B/L and *dr* was determined by ^1^H NMR, *dr* is the ratio of (±)-(*syn,anti*)-**3**/other diastereoisomers; Isolated yield. †T=50 ^o^C. ‡Solvent=THF. §OBoc of **2** was replaced with OP(OEt)_2_. ||The yield was determined by ^1^H NMR.

**Table 2:** The *B. subtilis* biofilm enhances the production of the anti-aging molecules NO and CSF.

**Media**	**Growth condition**	**NO production (NO**_**2**_^**−**^**+NO**_**3**_^**−**^ **μmol per 1.0 × 10**^**8**^ **cells)**	***csf–lacZ* activity (Miller Units per 1.0 × 10^8^ cells)**
MSgg	Planktonic	55.60±0.82	160.45±8.3
MSgg	Biofilm	257.60±25.20	540.50±21.1
NGM	Planktonic	180.00±19.10	225.83±15.2
NGM	Biofilm	1,160.00±45.20	1,740.15±75.1

Biofilms of the wild-type undomesticated strain NCIB3610 were developed in MSgg and NGM as indicated in the ‘Methods' section. After incubation for 48 h at 25 °C, the developed biofilms were disrupted as described previously^23^,^24^ and assayed for their NO-derivate species (nitrites and nitrates) content^28^ and CSF production levels, which are measured as levels of β-galactosidase activity driven by the promoter of the *csf* gene^62^. The average values of at least three independent experiments ±s.e.m. are presented.
