# Supplementary material for: Bacillus subtilis biofilm extends Caenorhabditis elegans longevity through downregulation of the insulin-like signalling pathway
Source: Nat Commun. 2017 Jan 30;8:14332. doi: 10.1038/ncomms14332 (PMC5290332; doi:10.1038/ncomms14332)
Supplement: Supplementary Information — Supplementary Figures and Supplementary Tables. [file ncomms14332-s1.pdf]

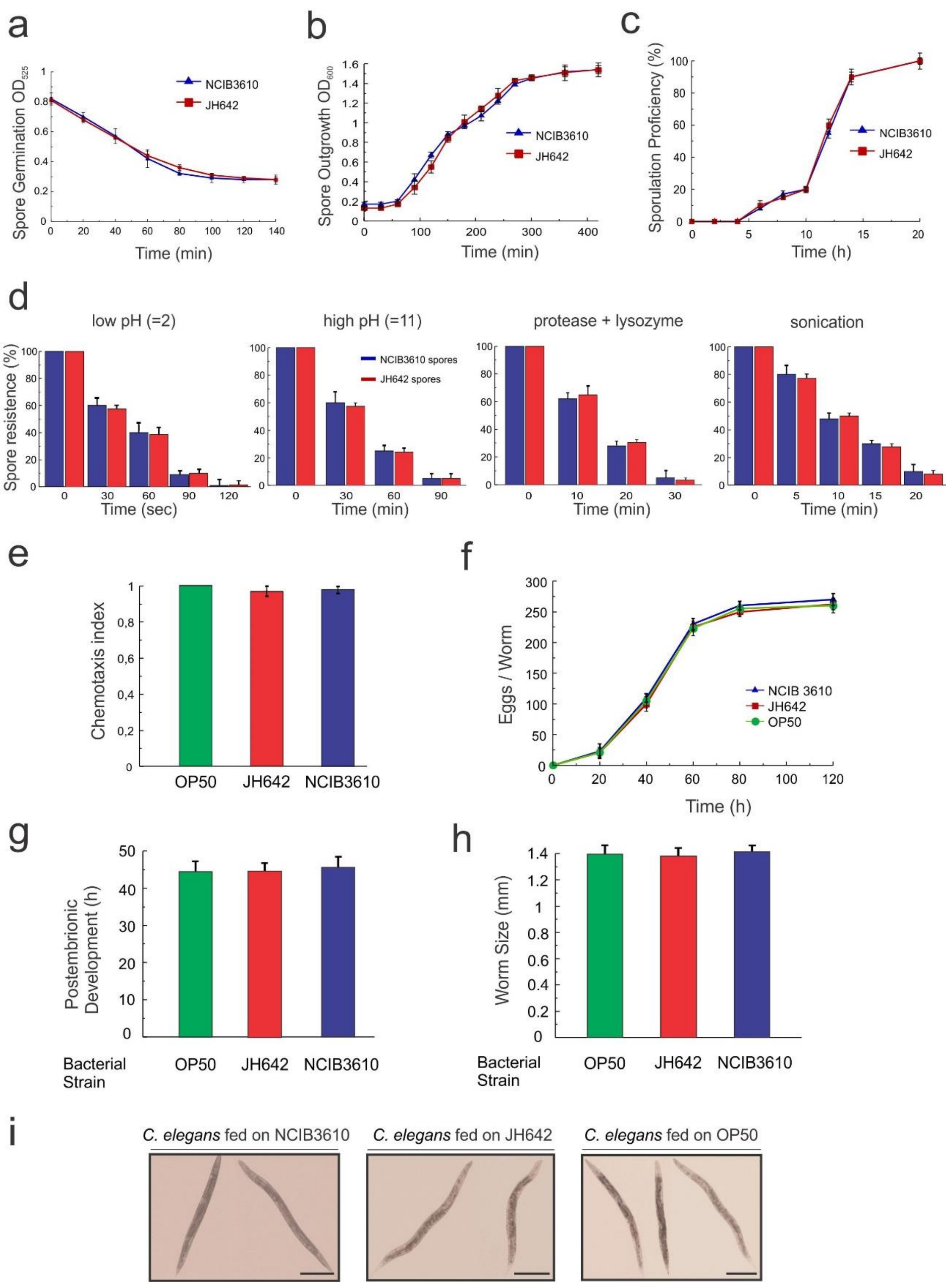

Supplementary Figure 1. Physiological parameters of bacterial growth and worm growth are not affected in spite of the use of different spore types. a) Germination rate of domesticated (JH642) and undomesticated (NCIB3610) *B. subtilis* spores in liquid NGM. Spore germination of JH642 (red squares) and NCIB3610 (blue triangles) *B. subtilis* was measured at 25 °C in presence of germination nutrients AFGK (10 mM L-alanine, 1 mM fructose, 1 mM glucose, and 10 mM potassium) as a decrease in optical density (loss of spore refractivity) at 525 nm. A representative experiment ± SEM performed in triplicate is shown. b) Outgrowth of JH642 and NCIB3610 *B. subtilis* spores is not affected in liquid NGM. Spore outgrowth of JH642 (red squares) and NCIB3610 (blue triangles) *B. subtilis* was monitored at 600 mm. NGM was

supplemented with 0.5% glucose for the growth of both types of vegetative cells after spore germination plus 0.01% tryptophan and 0.01% phenylalanine for the growth of JH642 *B. subtilis*, after germination. A representative experiment ± SEM performed in triplicate is shown. c) Sporulation proficiency of JH642 (red) and NCIB3610 (blue) cells44 over growth at 25 °C in NGM broth. A representative experiment ± SEM performed in triplicate is shown. d) Undomesticated and domesticated spores display similar physical, chemical and mechanic resistances against different stresses. Five milliliter of pure spore suspensions (1 x 10^5 CFU/mL) of the strains JH642 (domesticated) and NCIB3610 (undomesticated) were subjected to the different stresses shown in the graphs. Low and high pH values were reached with 1 M HCl and 0.5 M NaOH, respectively; protease + lysozyme treatment consisted of incubating the spores in the presence of proteinase K (1 mg/ml) plus lysozyme (1 mg/ml) and spore sonication was performed placing 0.2 µm glass beads and spores in a tube and subjecting it to sonication in a Branson 1201 sonicating water bath at 60 W and 67 kHz. All treatments were incubated at 37 °C with gentle shaking (50 rpm), and at the time indicated in each graph, 0.5 ml of spore suspension was taken, washed, and spread on LB agar plates to quantify spore survival as indicated in the Methods. Percentage (%) of spore survival is relative to the value at time zero (100 % of survival) for each treatment. A representative experiment ± SEM performed in triplicate is shown. e) Chemotactic behavior of N2 worms to *E. coli* or *B. subtilis* cells used as a food source. Briefly, age-synchronized late L4/young adult worms were transferred to plates containing 0.5 mm spots of OP50 *E. coli* or JH642 or NCIB3610 *B. subtilis*. The number of worms on each bacterial lawn was counted after 12 h of incubation at 20 °C, and the choice index was calculated as indicated in the “Methods”. The average of at least three independent experiments ± SEM is shown. OP50 *E. coli* (green) was used as a control. f) *B. subtilis* strains do not alter the eggs laying rate or amount. Worms were fed either on JH642 (red squares) or NCIB3610 (blue triangles) *B. subtilis* and transferred twice a day to prevent overcrowding until egg laying ceased. The progeny was counted 3 days after parents were removed, and their numbers are shown as the mean ± SEM from at least three experiments. OP50 *E. coli* (green circles) was used as a control. g) Domesticated and undomesticated *B. subtilis* do not affect postembryonic development. Eggs were allowed to hatch for 4 h and larvae were then placed individually on fresh NGM agar plates seeded with JH642 (red) or NCIB3610 (blue) *B. subtilis*. Worm growth was monitored every 5 h until they reached late L4/young adult stage. The mean number of h ± SEM from at least three experiments is shown. OP50 *E. coli* (green) was used as a control. h, i) Domesticated and undomesticated *B. subtilis* do not affect worm size. Bar graph (h) shows the average length ± SEM of 50 worms fed JH642 (red) or NCIB3610 (blue) *B. subtilis*. OP50 *E. coli* (green) was used as a control. (i) Worms were allowed to grow for an additional day after they reached late L4/young adult stage, and representative worms were photographed Scale bar = 300 µm.

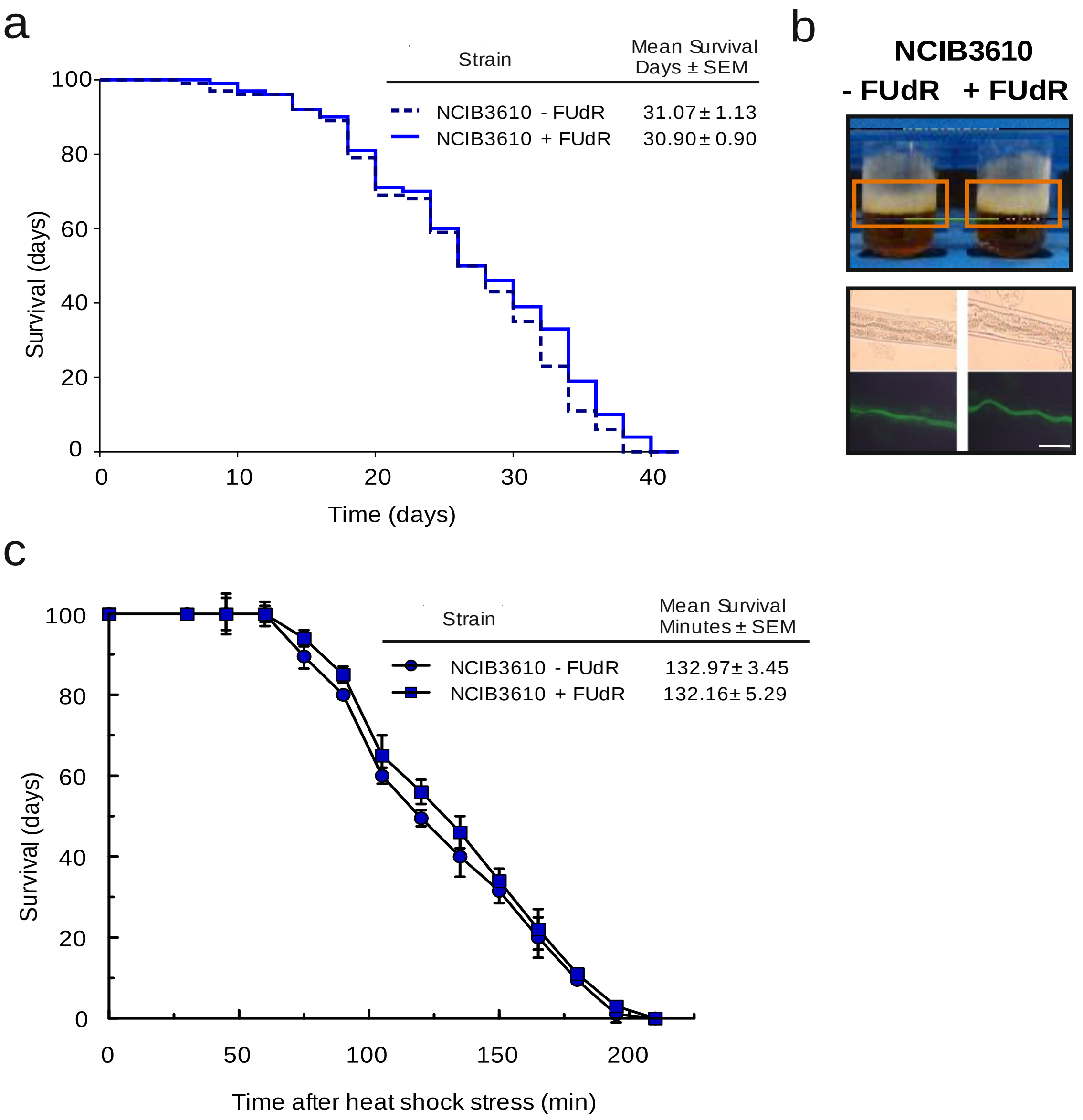

Supplementary Figure 2. The use of FUdR does not affect neither biofilm formation nor the lifespan and heat stress resistance of *C. elegans* conferred by undomesticated *B. subtilis*. a) Late L4/young adult N2 worms were fed on NCIB3610 *B. subtilis* cells on NGM agar plates in the complete absence of FUdR (blue dashed line) or in the presence of 16 µM FUdR (blue solid line) at 20 °C, and survival was monitored for signs of life as described in the "Methods" every 48 h. b) Biofilms of undomesticated *B. subtilis* strains were developed in liquid NGM in the presence or absence of 16 µM FUdR for 24 h at 25 °C. The area delimited by the rectangles corresponds to the biofilm (or pellicle) formed at the interface between the liquid and air. Images shown on the bottom are epifluorescence micrographs of typically stained N2 *C. elegans* fed on *epsA-gfp*-expressing NCIB3610 *B. subtilis* strain in the absence or in the presence of 16 µM FUdR. The fluorescence images were superimposed to differential interference contrast (DIC) images to depict the localization of the labels within the cells. Scale bar = 80 µm. c) Synchronized N2 worm NGM agar plates were fed on NCIB3610 *B. subtilis* cells in the complete absence of FUdR (blue circle) or in the presence of 16 µM FUdR (blue square) at 20 °C. As 5 days old adults, worm were shifted from 20 °C to 34 °C, and the mean survival was calculated as indicated in the "Methods".

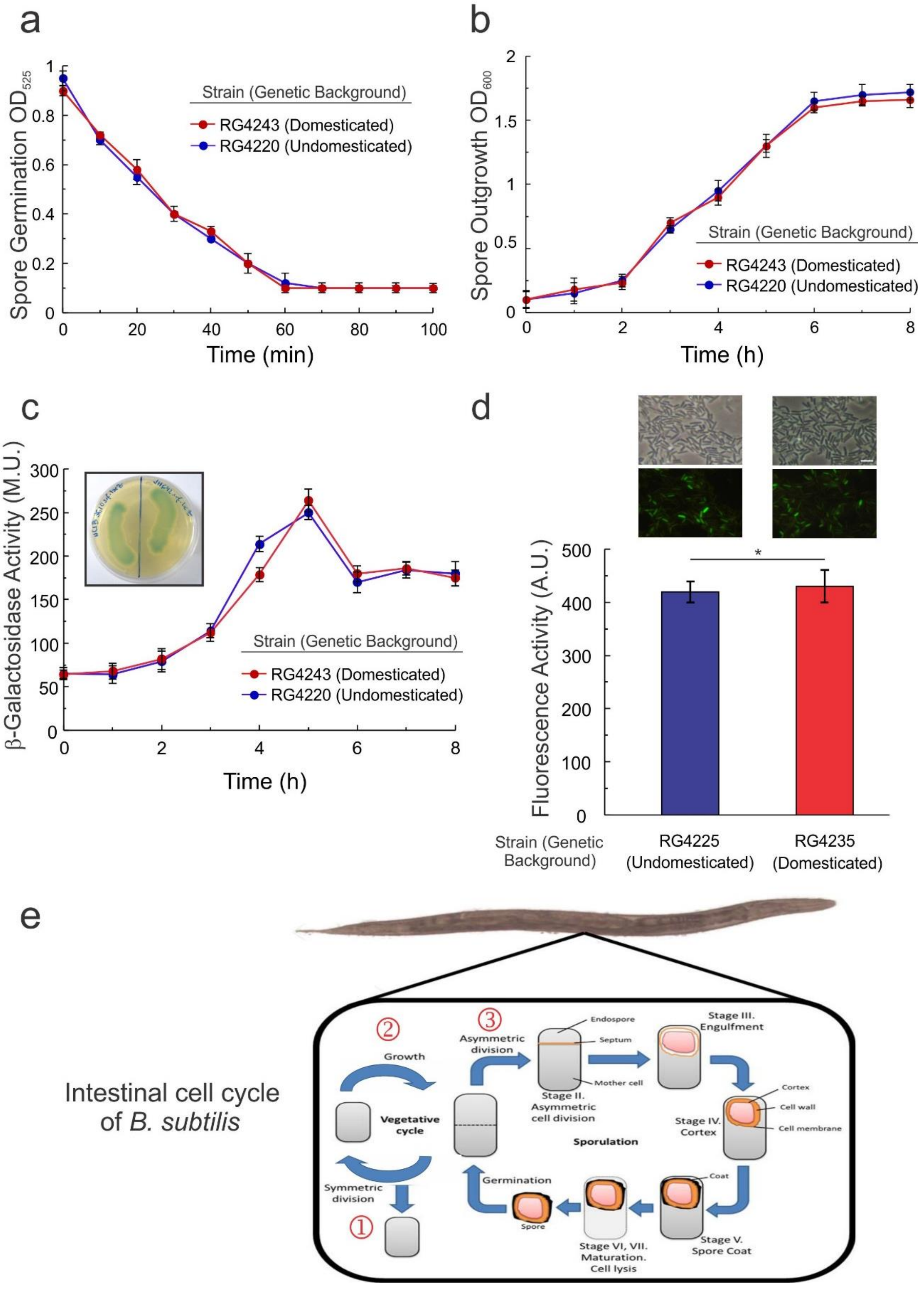

Supplementary Figure 3. a) Germination rate of spores of domesticated (RG4243) and undomesticated (RG4220) *B. subtilis*. Spore germination of RG4243 (red) and RG4220 (blue) *B. subtilis* was measured at 25 °C in the presence of the germination nutrients AFGK (10 mM L-alanine, 1 mM fructose, 1 mM glucose, and 10 mM potassium) as a decrease in optical density (loss of spore refractivity) at 525 nm. A representative experiment ± SEM performed in triplicate is shown. b) Outgrowth of domesticated (RG4243) and undomesticated (RG4220) *B. subtilis*

spores is not affected in liquid NGM. Spore outgrowth of RG4220 (blue) and RG4243 (red) *B. subtilis* was monitored at 600 mm. Shown is a representative experiment ± SEM from triplicate. c) β-galactosidase expression driven by the P*srf-lacZ* construct harbored in domesticated and undomesticated *B. subtilis*. Cultures of domesticated (RG4243, red) and undomesticated (RG4223, blue) *B. subtilis* cells were developed in liquid NGM at 25 °C with shaking. Every 12 h, 20 worms were taken, washed and disrupted. Determination of β-galactosidase activity was performed using worm extracts. The β-galactosidase activity, expressed as Miller units (MU) over time (h) is shown. The insert photograph shows the growth and β-galactosidase expression (blue color) of strains RG4220 (right) and RG 4243 (left) after 30 h of incubation at 25 °C in NGM-Xgal agar plate. After the cells were incubated, the cells were removed, resuspended in Z-buffer at a final concentration of 2 x 10^7 UFC/ml and assayed for β-galactosidase activity. The levels of β-galactosidase activity of strains RG4220 and RG4243 grew on solid NGM medium were 1,350 ± 115 MU and 1,425 ± 130 MU, respectively. d) Fluorescence activity driven by domesticated and undomesticated *B. subtilis* strains harboring an ectopically integrated *epsA-gfp* fusion (Supplementary Table 1). The bacteria were planktonically grew at 25 °C on NGM agar plates supplemented with 0.5% glucose for the growth of both types of cells (RG4225 and RG4235) plus 0.01% tryptophan and 0.01% phenylalanine. After 24 h, bacteria were taken, washed resuspended and imaged by fluorescence microscopy as indicated in the Methods. Fluorescence intensity is indicated as arbitrary units (A.U.) per 1 x 10^5 bacterial cells, and error bars show the mean ± SEM from at least three independent experiments. The inserts shown are epifluorescence micrographs of domesticated- and undomesticated *epsA-gfp* cells grown on solid NGM plates. Scale bar = 3.0 μm. *P < 0.05 (ANOVA with Bonferroni test). e) Worm intestinal cell cycle of *B. subtilis*. After spore germination (1), bacterial cells proliferate by vegetative cell division (symmetric division) that produces identical sister cells and growth (2). Starvation initiates sporulation where an asymmetric cell division (3) produces two cells of different size and cell destiny, the smaller forespore cell and the larger mother cell. The mother cell encloses and nurtures the forespore, deposits the protective spore coat, and eventually dies by lysis. The mature spore is released when the mother cell lyses and germinates under favorable conditions to start a new division cycle.

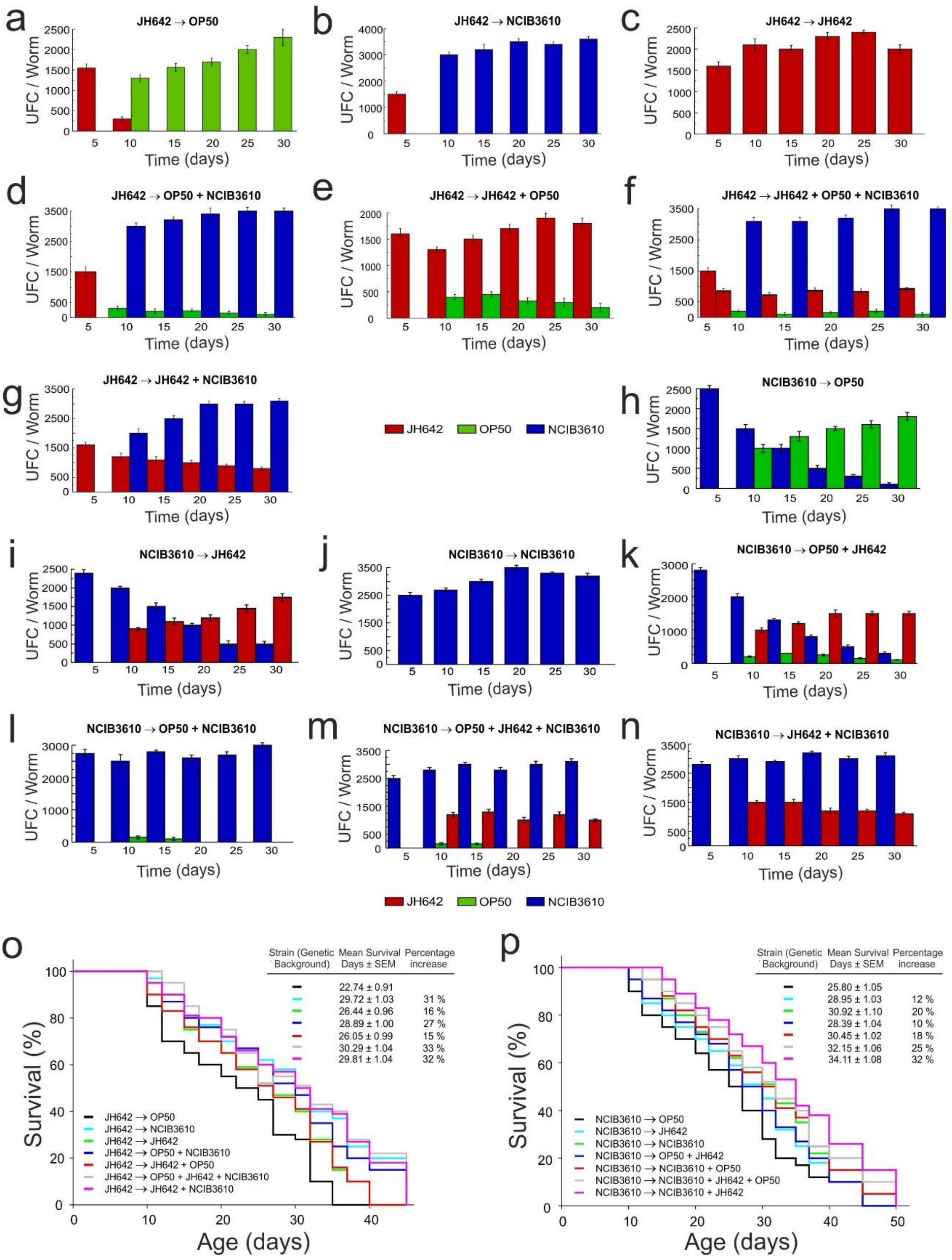

Supplementary Figure 4. Worm gut colonization and persistence. A large population of young *C. elegans* N2 (late L4/young adult stage) was synchronized and fed on a lawn of JH642 (graphs a-g) or NCIB3610 (graphs h-n) spores for five days. After this time (time "5" in the graphs), worms were washed and placed on 10 mm NGM agar plates containing either a lawn of spores of JH642 or NCIB3610 strains or OP50 cells or mixtures of OP50 + NCIB3610 or JH642 + OP50 or JH642 + OP50 + NCIB3610 cells as indicated in each graph and incubated at 20 °C. After the indicated incubation period, 100 worms were taken and disrupted as indicated in the Methods to quantify the number of bacterial cells of each type of strain present in the worm intestine. The differentiation between OP50, JH642 and NCIB3610 colonies (in the case of the use of bacterial

mixtures as worm food source) was established in the big differences in colony architecture between them. In addition to measuring bacterial gut colonization/persistence, worm survival (o-p) was measured as indicated in the “Methods”. The presented results are representative of at least three independent experiments ± SEM.

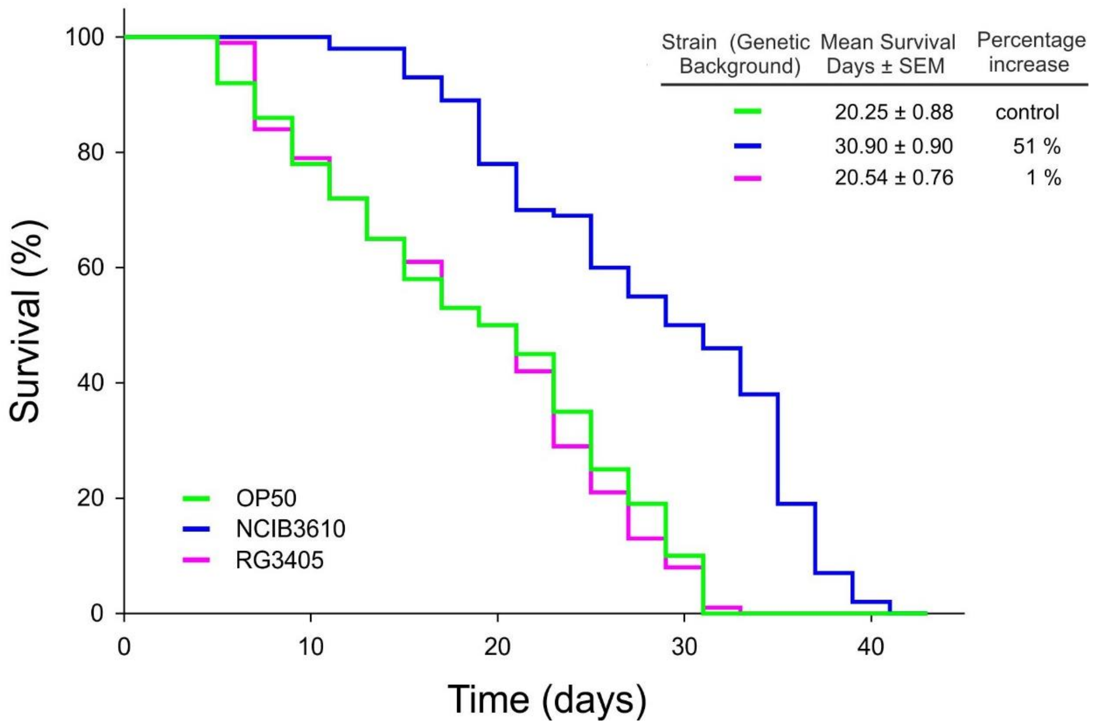

Supplementary Figure 5. Longevity of *C. elegans* fed on *B. subtilis* cells deficient in biofilm formation and CSF production. Late L4/young adult N2 worms were fed on spores of RG3405 *B. subtilis* cells (*ΔbslAΔcsf*) on NGM agar plates at 20 °C, and survival was monitored every 48 h. The mean survival was calculated as indicated in the "Methods". Worms fed on OP50 *E. coli* (green) and NCIB3610 (blue) *B. subtilis* were used as a control. The figure shows the average of at least three independent experiments ± SEM, and the survival increase is expressed as the total number of worms fed on OP50 *E. coli*.

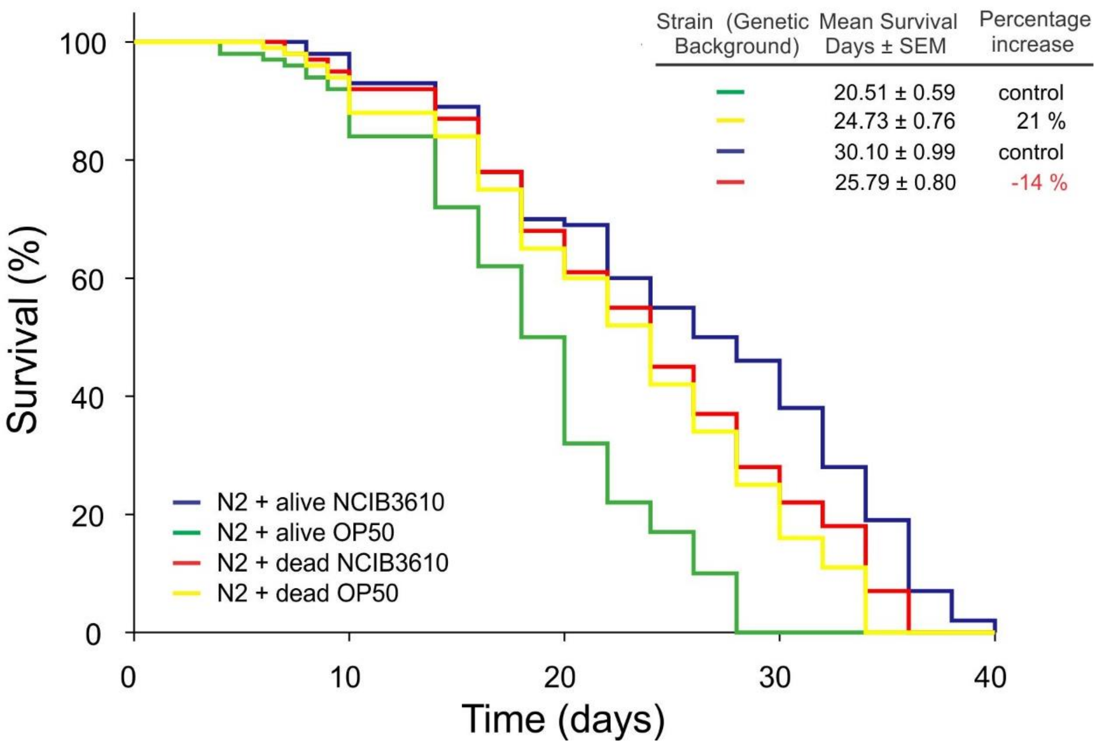

Supplementary Figure 6. Effects of alive and dead OP50 *E. coli* and NCIB3610 *B. subtilis* in *C. elegans* lifespan. Late L4/young adult N2 worms were fed either on alive (green) or dead (yellow) OP50 *E. coli* or alive (blue) or dead (red) NCIB3610 *B. subtilis* cells on NGM agar plates at 20 °C, and survival was monitored for signs of life as described in the "Methods" every 48 h. To obtain dead OP50 cells, the bacterial culture was heat-treated for 20 minutes at 80 °C to kill vegetative cells. To obtain dead NCIB3610 cells, the bacterial culture was heat-treated for 30 minutes at 121 °C (autoclaved) to kill spores. A representative experiment of at least three independent experiments ± SEM is shown.

Bacterial strains	Relevant phenotype and/or genotype	Reference and/or source
OP50	*E. coli* strain commonly used to feed *C. elegans*	Caenorhabditis Genetic Center, CGS
JH642	*B. subtilis* domesticated strain	Laboratory collection (52)
NCIB3610	*B. subtilis* Marburg, undomesticated strain	Laboratory collection (49)
RG4365	*B. subtilis* natto, undomesticated strain	Laboratory collection (43)
RG4243	JH642 *srf-lacZ::amyE*-cm^r	Laboratory collection (39)
RG4220	NCIB3610 *srf-lacZ::amyE*-cm^r	This work, RG4243→NCIB3610 (DNA from donor strain → receptor strain)
NRS2289	*sacA::PbslA-gfpmut2*-kn^r	Laboratory collection (38)
YC164	pNGFP-*PespA-gfp*-cm^r	Laboratory collection (38)
RG4225	NCIB3610 *sacA::PbslA-gfpmut2*-kn^r	This work, NRS2289→NCIB3610
RG4235	JH642 *sacA::PbslA-gfpmut2*-kn^r	This work, NRS2289→JH642
RG3601	NCIB3610 Δ*tasA::*spc^r	Laboratory collection (26)
RG3602	NCIB3610 Δ*epsG::*pBL601-spc^r	Laboratory collection (26)
RG3603	NCIB3610 Δ*bslA::*phe^r	Laboratory collection (26)
RG3000	168 Δ*nos::*ery^r	Bacillus Genetic Stock Center (BGSC)
RG3610	NCIB3610 Δ*nos::*ery^r	This work, RG3000→NCIB3610
RG3620	NCIB3610 pNGFP-*PespA-gfp*-cm^r	YC164→NCIB3610
RG3621	NCIB3610 Δ*nos::*ery^r pNGFP-*PespA-gfp*-cm^r	YC164→RG3610
RG3622	NCIB3610 Δ*bslA::*tc^r pNGFP-*PespA-gfp*-cm^r	YC164→RG3603
RG3611	NCIB3610 Δ*nos::*ery^r /Δ*bslA::*phe^r	This work, RG3603→RG3610
RG3623	NCIB3610 Δ*nos::*ery^r /Δ*bslA::*phe^r pNGFP-*PespA-gfp*-cm^r	This work, YC164→RG3611
RG657	JH642 Δ*csf::*tc^r	Laboratory collection (102)
RG4010	NCIB3610 Δ*csf::*tc^r	This work, RG657→NCIB3610
RG3405	NCIB3610 Δ*bslA::*phe^r /Δ*csf::*tc^r	This work, RG4010→RG3603
LC3601	NCIB3610 Δ*nos::*ery^r /Δ*csf::*tc^r	This work, RG657→RG3610

LC3611	NCIB3610 Δ*nos*::tc^r/Δ*bslA*::phe^r/Δ*csf*::spc^r	This work, RG4010 → RG3611
***C. elegans* strains**	**Relevant genotype**	**Reference and/or source**
N2	Reference wt strain, isolated in Bristol, U.K.	Caenorhabditis Genetic Center, CGS
CB1370	*daf-2 (e1370)III*	Caenorhabditis Genetic Center, CGS
CF1038	*daf-16 (mu86) I*	Caenorhabditis Genetic Center, CGS
PS3551	*hsf-1 (sy441) I*	Caenorhabditis Genetic Center, CGS

Supplementary Table 1. Bacterial and *C. elegans* strains used in this work.

C. elegans strain	Related to figure	Type of stress	Bacterial strain (genetic background)	Repeats	Number of animals that died/total	Mean survival ([a]days or [b]minutes)	Average of mean survivals ± S.E.M. ([a]days or [b]minutes)	Interval of average survival with 95 % ([a]days or [b]minutes)	Average survival increase (%) / P value
N2	1a	None	OP50 (wt)	1	80/90	18.33 [a]	20.25 ± 0.88 [a]	18.52 - 21.97 [a]	control
				2	98/100	21.53 [a]			
				3	99/99	20.89 [a]			
			JH642 (wt)	1	89/98	24.95 [a]	25.02 ± 0.83 [a]	23.39 - 26.65 [a]	23.56 / 0.0004
				2	93/100	26.05 [a]			
				3	97/100	24.06 [a]			
			NCIB3610 (wt)	1	89/100	31.27 [a]	30.90 ± 0.90 [a]	29.14 - 32.67 [a]	52.59 / 0
				2	87/100	30.95 [a]			
				3	94/100	30.48 [a]			
	1b	Temperature (34 ºC)	OP50	1	50/50	34.50 [b]	34.91 ± 1.04 [b]	32.88 - 36.94 [b]	control
				2	60/60	35.25 [b]			
				3	61/61	34.97 [b]			
			JH642	1	55/55	97.64 [b]	97.58 ± 2.03 [b]	93.59 - 101.57 [b]	179.52 / 0
				2	60/60	97.5 [b]			
				3	57/57	97.6 [b]			
			NCIB3610	1	55/55	131.18 [b]	132.16 ± 5.29 [b]	121.81 - 142.51 [b]	278.57 / 0
				2	50/50	133.20 [b]			
				3	52/53	132.10 [b]			
	1c	Osmotic (NaCl 200 mmol/L)	OP50	1	74/74	120.43 [b]	114.43 ± 6.11 [b]	102.46 - 126.41 [b]	control
				2	75/76	116.90 [b]			
				3	70/70	105.96 [b]			
			JH642	1	91/91	201.85 [b]	206.79 ± 8.35 [b]	190.42 - 223.16 [b]	80.71 / 0
				2	85/85	209.56 [b]			
				3	92/94	208.96 [b]			
			NCIB3610	1	92/92	253.80 [b]	257.96 ± 4.49 [b]	249.16 - 266.75 [b]	125.43 / 0
				2	94/96	251.64 [b]			

				3	90/90	268.44 [b]			
	1d	Metal (Cd^{2+} 50 μmol/L)	OP50	1	71/71	45.13 [b]	46.66 ± 3.08 [b]	40.62 - 52.70 [b]	control
				2	65/65	47.76 [b]			
				3	69/69	47.09 [b]			
			JH642	1	108/108	79.78 [b]	80.18 ± 4.60 [b]	71.15 - 89.20 [b]	71.75 / 0.00000001
				2	110/110	73.88 [b]			
				3	107/107	86.88 [b]			
			NCIB3610	1	80/81	107.43 [b]	110.38 ± 5.18 [b]	100.23 - 120.53 [b]	136.56 / 0
				2	78/78	109.08 [b]			
				3	75/75	114.63 [b]			
	1e	Oxidative (H_2O_2 25 mmol/L)	OP50	1	61/61	78.56 [b]	81.00 ± 3.69 [b]	73.77 - 88.23 [b]	control
				2	62/62	83.46 [b]			
				3	60/60	80.98 [b]			
			JH642	1	71/71	109.12 [b]	109.50 ± 4.36 [b]	100.96 - 118.04 [b]	35.19 /0.0000008
				2	70/70	104.85 [b]			
				3	71/71	114.53 [b]			
			NCIB3610	1	74/74	133.26 [b]	139.80 ± 5.25 [b]	129.51 - 150.09 [b]	72.59 / 0
				2	80/81	142.69 [b]			
				3	78/78	143.45 [b]			

Supplementary Table 2: Lifespan analysis related to Figure 1.

Undomesticated *B. subtilis* cells extend *C. elegans* survival under normal and stressful (thermal, osmotic, metal and oxidative) conditions. Each data set consists of three independent repeats fitted to Boltzmann sigmoid curve and the mean survival time ± SEM and the confidence interval (CI) are indicated. Percentage (%) survival change was determined against control OP50 *E. coli* cells under the same experimental condition (type of stress). Independent experimental and control analysis were done side by side and indicated by same number (1, 2, or 3) in the repeat column. *P* -values for each treatment were calculated against the correspondent control by Log-rank test. Survival data were calculated using Kaplan-Meier test.

C. elegans strain	Related to figure	Type of stress	Bacterial strain	FUdR	Repeats	Number of dead animals/total	Mean survival (days[a]/minutes[b])	Average of mean survivals ± S.E.M. (days[a]/minutes[b])	Interval of average survival with 95% (days[a]/minutes[b])	Average survival increase (%)
N2	S2a	None	NCIB3610	-	1	99/100	30.88[a]	31.07 ± 1.13[a]	29.45 - 32.78[a]	53.43
				-	2	98/100	31.36[a]			
				-	3	93/100	31.29[a]			
N2			NCIB3610	+	1	89/100	31.27[a]	30.90 ± 0.90[a]	29.14 - 32.67[a]	52.59
				+	2	87/100	30.95[a]			
				+	3	94/100	30.48[a]			
N2	S2c	Temperature	NCIB3610	-	1	62/62	132.88[b]	132.97 ± 3.45[b]	124.52 - 143.48[b]	280.89
				-	2	51/53	132.35[b]			
				-	3	50/50	133.69[b]			
N2			NCIB3610	+	1	55/55	131.18[b]	132.16 ± 5.29[b]	121.81 - 142.51[b]	278.57
				+	2	50/50	133.20[b]			
				+	3	52/53	132.10[b]			

Supplementary Table 3. Undomesticated *B. subtilis* cells extend *C. elegans* survival and confer resistance to heat shock either in the presence or in the absence of FUdR. Each data set consists of three independent repeats fitted to Boltzmann sigmoid curve and the mean survival time ± SEM and the confidence interval (CI) are indicated. Percentage (%) survival change was determined against control OP50 *E. coli* cells under the same experimental condition (type of stress). Independent experimental and control analysis were done side by side and indicated by same number (1, 2, or 3) in the repeat column. Survival for each treatment was calculated against the correspondent control using Kaplan-Meier test.

C. elegans strain	Relatived to supplementary figure	First bacterial strain	Second bacterial strain (genetic background)	Repeats	Number of animals that died/total	Mean survival days	Average of mean survival days ± S.E.M	Interval of average survival days with 95 %	Average survival increase (%)
N2	4o	JH642	OP50 (wt)	1	100/100	26.79	22.74 ± 0.91	20.96 - 24.52	control
				2	92/100	19.43			
				3	100/100	22.00			
			JH642 (wt)	1	100/100	25.28	26.44 ± 0.96	24.56 - 28.32	16.27
				2	99/100	26.88			
				3	100/100	27.16			
			NCIB3610 (wt)	1	100/100	30.16	29.72 ± 1.03	27.70 - 31.74	30.69
				2	98/100	29.54			
				3	100/100	29.46			
			OP50 + NCIB3610	1	100/100	30.26	28.89 ± 1.00	26.93 - 30.86	27.05
				2	100/100	29.07			
				3	100/100	27.34			
			OP50 + JH642	1	96/100	26.42	26.05 ± 0.99	24.12 - 27.98	14.56
				2	100/100	26.00			
				3	100/100	25.73			
			OP50 + JH642 + NCIB3610	1	100/100	29.40	30.29 ± 1.04	28.25 - 32.34	33.20
				2	100/100	31.86			
				3	100/100	29.61			
			JH642 + NCIB3610	1	95/100	27.60	29.81 ± 1.04	27.77 - 31.85	31.91
				2	100/100	31.09			
				3	100/100	31.28			
			OP50	1	100/100	26.43	25.80 ± 1.05	23.74 - 27.86	control
				2	100/100	24.64			
				3	98/100	26.33			
			JH642	1	100/100	30.00	28.95 ± 1.03	26.94 - 30.97	12.21
				2	100/100	28.53			
				3	100/100	28.32			
			NCIB3610	1	100/100	30.62	30.92 ± 1.1	28.76 - 33.08	19.84
				2	97/100	33.29			
				3	100/100	28.85			
			OP50 +	1	100/100	29.64			

	4p	NCIB3610	OP50 + JH642	2	100/100	26.83	28.39 ± 1.04	26.35 - 30.43	10.04
				3	95/100	28.70			
			OP50 + NCIB3610	1	100/100	31.26	30.45 ± 1.02	28.45 - 32.46	18.02
				2	100/100	29.21			
				3	100/100	30.88			
			OP50 + JH642 + NCIB3610	1	98/100	33.56	32.15 ± 1.06	30.08 - 34.22	24.61
				2	100/100	35.26			
				3	100/100	27.63			
			JH642 + NCIB3610	1	100/100	35.12	34.11 ± 1.08	32 - 36.22	32.21
				2	100/100	33.26			
				3	100/100	33.95			

Supplementary Table 4. Lifespan analysis related to Supplementary Figure 4.
Undomesticated *B. subtilis* cells are better suited to colonize and persist in the gut of *C. elegans*. Each data set consists of three independent repeats fitted to Boltzmann sigmoid curve and the mean survival time ± SEM and CI are indicated. Percentage (%) survival change was determined against control worms fed on JH642 or NCIB3610 *B. subtilis* cells and shifted to the presence of OP50 *E. coli* cells. Independent experimental and control analysis were done side by side and indicated by same number (1, 2, or 3) in the repeat column. Survival data were calculated by Kaplan-Meier test.

C. elegans strain	Relatived to figure	Bacterial strain (genetic background)	Repeats	Number of animals that died/total	Mean survival days	Average of mean survival days ± S.E.M.	Interval of average survival days with 95 %	Average survival increase (%)
N2	3b	OP50 (wt)	1	80/90	18.33	19.53 ± 0.93	17.70 - 21.36	control
			2	79/95	21.36			
			3	88/93	18.90			
		NCIB3610 (wt)	1	96/105	30.68	31.00 ± 0.89	29.25 - 32.75	58.73
			2	99/106	31.52			
			3	94/101	30.80			
		RG3601 (Δ*tasA*)	1	91/100	20.35	21.84 ± 0.95	19.98 - 23.71	11.82
			2	93/99	22.01			
			3	99/101	23.16			
		RG3602 (Δ*epsG*)	1	89/100	22.86	22.90 ± 0.83	21.28 - 24.52	17.26
			2	95/100	22.78			
			3	98/101	23.06			
		RG3603 (Δ*bslA*)	1	89/100	23.76	23.26 ± 1.07	21.16 - 25.36	19.10
			2	97/101	22.79			
			3	98/100	23.23			
	3d	OP50	1	82/95	18.35	19.45 ± 0.63	17.62 - 21.28	control
			2	90/95	21.05			
			3	92/98	18.95			
		NCIB3610	1	103/105	32.06	31.28 ± 0.88	29.08 - 33.42	60.82
			2	92/102	30.68			
			3	98/104	31.10			
		RG3601 + RG3602 + RG3603	1	88/95	30.05	29.59 ± 1.05	27.52 - 31.65	52.13
			2	98/99	29.48			
			3	99/100	29.24			

Supplementary Table 5. Lifespan analysis related to Figure 3.

Bacillus subtilis biofilm formation proficiency is required to extend the longevity of *C. elegans*. Each data set consists of three independent repeats fitted to Boltzmann sigmoid curve and the mean survival time ± SEM and CI are indicated. Percentage (%) survival change of worms fed on wild-type or biofilm-deficient *B. subtilis* cells was determined against control worms fed on OP50 *E. coli* cells. Independent experimental and control analysis were done side by side and indicated by same number (1, 2, or 3) in the repeat column. Survival data were calculated by Kaplan-Meier test.

MEDIA	BACTERIAL STRAIN	GROWTH CONDITION	NO production (nitrate and nitrite mmol /1.0 X 10^8 cells)
NGM	OP50	Aerobic	5.2 ± 0.7
NGM	OP50	Microaerophilic	132.5 ± 0.9
NGM	OP50	Anaerobic	24.9 ± 0.8

Supplementary Table 6. NO production proficiency of OP50 *E. coli* cells grown under different oxygen tensions. The OP50 strain was grown with shaking (180 rpm, aerobic condition), without shaking (microaerophilic condition) or in anaerobic jar (Oxoid-anaerogen 2.5L, anaerobic condition) in NGM broth at 25 °C for 36 h. Then, the supernatants of each culture were taken and filter sterilized. Appropriate aliquots of each cell-free supernatant were assayed for the presence of NO-derived metabolites (NO_2^- and NO_3^-). Data are representative of at least three experiments made by triplicate.

C. elegans strain	Relatived to figure	Bacterial strain (genetic background)	Repeats	Number of animals that died/total	Mean survival days	Average of mean survival days + S.E.M.	Interval of average survival days with 95 %	Average survival increase (%)
N2	4a	OP50 (wt)	1	101/106	20.16	19.78 ± 0.94	17.93 - 21.63	control
			2	104/405	18.88			
			3	100/100	20.30			
		NCIB3610 (wt)	1	92/92	29.86	30.29 ± 0.88	28.26 - 32.33	53.13
			2	89/95	31.29			
			3	93/94	29.72			
		RG3610 (Δ*nos*)	1	78/102	24.82	25.55 ± 1.14	23.31 - 27.79	29.17
			2	98/100	26.98			
			3	89/104	24.82			
		RG3603 (Δ*bslA*)	1	102/102	22.08	22.03 ± 0.97	20.13 - 23.94	11.38
			2	100/103	21.38			
			3	98/99	22.63			
		RG3611 (Δ*nos* Δ*bslA*)	1	92/100	20.48	20.06 ± 0.79	18.52 - 21.60	1.42
			2	91/100	20.24			
			3	96/100	19.46			
		RG3610 + RG3603	1	92/98	29.73	29.51 ± 1.14	27.27 - 31.75	49.19
			2	89/96	29.47			
			3	92/100	29.33			

Supplementary Table 7. Lifespan analysis related to Figure 4.

Biofilm formation proficiency and NO production contribute to extend *C. elegans* longevity. Each data set consists of three independent repeats fitted to Boltzmann sigmoid curve and the mean survival time ± SEM and CI are indicated. Percentage (%) survival change of worms fed on wild-type or mutant (Δ*nos*, Δ*bslA*, Δ*nos* Δ*bslA*, or Δ*nos* + Δ*nos* Δ*bslA*) *B. subtilis* cells was determined against control worms fed on OP50 *E. coli* cells. Independent experimental and control analysis were done side by side and indicated by same number (1, 2, or 3) in the repeat column. Survival data were calculated by Kaplan-Meier test.

C. elegans strain	Relative to figure	Bacterial strain (genetic background)	Repeats	Number of animals that died/total	Mean survival days	Average of mean survival days ± S.E.M.	Interval of average survival days with 95 %	Average survival increase (%)
N2	5a	OP50 (wt)	1	100/100	19.65	20.14 ± 0.82	18.53 - 21.75	control
			2	87/100	22.06			
			3	100/100	18.71			
		NCIB3610 (wt)	1	100/102	31.06	30.74 ± 0.94	28.9 - 32.58	52.63
			2	96/100	30.16			
			3	91/100	31.00			
		RG4010 (Δ*csf*)	1	100/103	25.86	26.16 ± 0.89	24.41 - 27.91	29.89
			2	100/100	27.59			
			3	100/105	25.03			
		LC3601 (Δ*nos* Δ*csf*)	1	95/100	21.84	23.85 ± 0.92	22.05 - 25.65	18.42
			2	85/100	25.06			
			3	93/100	24.65			
		LC3611 (Δnos Δ*bslA* Δ*csf*)	1	92/100	19.68	18.79 ± 0.63	17.55 - 20.03	- 6.70
			2	96/100	17.99			
			3	99/100	18.70			
		RG3611 (Δ*nos* Δ*bslA*) + RG4010 (Δ*csf*)	1	98/100	29.68	29.68 ± 1.06	27.59 - 31.77	47.36
			2	99/100	28.64			
			3	100/100	30.72			
	5b	OP50 (wt)	1	98/101	20.02	18.68 ± 0.63	17.44 - 19.92	control
			2	95/100	18.81			
			3	100/100	17.21			
		NCIB3610 (wt)	1	100/100	27.98	30.10 ± 0.99	28.15 - 32.05	61.13
			2	98/100	31.15			
			3	101/101	31.17			
		OP50 + CSF	1	98/102	24.27	23.98 ± 0.94	22.14 - 25.82	28.37
			2	96/100	23.64			
			3	101/104	24.03			
	5c	LC3611	1	100/103	17.15	16.06 ± 1.09	13.93 - 18.19	control
			2	101/101	16.05			
			3	105/105	14.98			
		RG3611	1	99/100	16.85	17.52 ± 0.94	15.55 - 19.49	9.09
			2	96/100	17.38			
			3	100/100	18.33			
		LC3611 + CSF	1	100/101	19.26	20.02 ± 1.00	18.48 - 21.56	24.66
			2	95/100	21.65			
			3	99/100	19.15			

Supplementary Table 8. Lifespan analysis related to Figure 5.
The quorum-sensing pentapeptide CSF extends *C. elegans* longevity. Wild-type and isogenic *B. subtilis* mutants strains (Δ*bslA*, Δ*nos*, Δ*csf*, Δ*nosΔcsf*, or Δ*nos* Δ*bslAΔcsf*) were used to feed wild-type N2 worms. Each data set consists of three independent repeats fitted to Boltzmann sigmoid curve and the mean survival time ± SEM and CI are indicated. Percentage (%) survival change was determined against control OP50 *E. coli* (5a,b) or LC3611 *B. subtilis* (5c) cells, respectively. Independent experimental and control analysis were done side by side and indicated by same number (1, 2, or 3) in the repeat column. Survival data for each treatment, including endogenous (bacterial, 5a) or exogenous CSF treatments (5b,c), were calculated against the correspondent control using Kaplan-Meier test.

C. elegans strain (genetic background)	Bacterial strain (genetic background)	FUdR	Repeats	Number of dead animals/total	Mean survival (days)	Average of mean survivals ± S.E.M. (days)	Interval of average survival with 95% (days)	Average survival increase (%)
N2 (wt)	NCIB3610	-	1	99/100	30.88	31.07 ± 1.13	29.45 - 32.78	control
		-	2	98/100	31.36			
		-	3	93/100	31.29			
	NCIB3610	+	1	89/100	31.27	30.90 ± 0.90	29.14 - 32.67	- 0.55
		+	2	87/100	30.95			
		+	3	94/100	30.48			
	RG3603 (Δ*bslaA*)	-	1	99/100	23.76	23.26 ± 2.10	21.16 - 25.36	control
		-	2	98/100	22.79			
		-	3	93/100	23.23			
	RG3603 (Δ*bslaA*)	+	1	87/90	22.98	23.41 ± 1.19	22.22 - 24.60	0.65
		+	2	57/59	23.56			
		+	3	63/65	23.69			
	RG3610 (Δ*nos*)	-	1	87/90	25.98	26.19 ± 1.19	22.22 - 24.60	control
		-	2	57/59	26.56			
		-	3	63/65	26.69			
	RG3610 (Δ*nos*)	+	1	78/102	26.04	26.38 ± 2.29	24.82 - 29.4	0.73
		+	2	98/100	27.11			
		+	3	89/104	25.98			
	RG4010 (Δ*csf*)	-	1	62/62	27.38	27.18 ± 2.45	25.29 - 30.19	control
		-	2	51/53	27.02			
		-	3	50/50	27.14			
	RG4010 (Δ*csf*)	+	1	87/102	26.98	27.14 ± 2.18	24.95 - 29.32	- 0.15
		+	2	99/99	27.11			
		+	3	99/105	27.33			
CF1038 (*daf-16*)	NCIB3610	-	1	78/78	17.17	17.17 ± 1.45	15.72 - 18.62	control
		-	2	82/82	17.23			
		-	3	81/81	17.12			
	NCIB3610	+	1	98/100	17.37	17.34 ± 1.98	15.85 - 18.74	0.99
		+	2	72/75	17.44			
		+	3	89/102	17.22			
	RG3603 (Δ*bslaA*)	-	1	62/62	14.85	14.69 ± 1.45	14.12 - 15.36	control
		-	2	51/53	14.98			
		-	3	50/50	14.25			
	RG3603 (Δ*bslaA*)	+	1	86/99	13.95	14.91 ± 0.87	14.04 - 15.78	0.15
		+	2	80/99	14.65			
		+	3	100/100	16.13			
	RG3610 (Δ*nos*)	-	1	79/82	26.25	26.41 ± 1.44	29.45 - 32.78	control
		-	2	68/68	26.98			

		-	3	73/80	25.99			
	RG3610 (Δ*nos*)	+	1	86/87	26.08	26.33 ± 1.76	24.57 - 28.09	- 0.30
		+	2	95/95	26.48			
		+	3	86/87	26.43			
	RG4010 (Δ*csf*)	-	1	98/100	26.78	26.82 ± 2.45	25.02 - 28.68	control
		-	2	95/95	26.56			
		-	3	50/52	27.12			
	RG4010 (Δ*csf*)	+	1	89/92	26.34	26.80 ± 1.84	24.96 - 28.64	- 0.075
		+	2	95/95	26.98			
		+	3	105/102	27.08			
PS3551 (*hsf-1*)	NCIB3610	-	1	100/106	17.45	17.47 ± 1.34	16.45 - 17.78	control
		-	2	95/100	17.97			
		-	3	102/102	16.99			
	NCIB3610	+	1	100/100	17.44	17.35 ± 1.17	16.18 - 18.52	- 0.68
		+	2	100/101	16.05			
		+	3	97/99	18.56			
	RG3603 (Δ*bslaA*)	-	1	62/62	14.35	14.68 ± 1.45	13.89 - 15.28	control
		-	2	51/53	14.89			
		-	3	50/50	14.80			
	RG3603 (Δ*bslaA*)	+	1	100/102	15.18	14.72 ± 1.07	13.65 - 15.79	0.27
		+	2	97/100	14.69			
		+	3	100/100	14.29			
	RG3610 (Δ*nos*)	-	1	99/100	16.58	16.36 ± 2.03	15.10 - 17.65	control
		-	2	98/99	16.39			
		-	3	101/101	16.10			
	RG3610 (Δ*nos*)	+	1	100/106	18.49	16.21 ± 1.23	14.98 - 17.44	- 0.92
		+	2	95/100	15.94			
		+	3	102/102	15.20			
	RG4010 (Δ*csf*)	-	1	74/74	16.89	16.47 ± 1.40	15.52 - 17.48	control
		-	2	77/78	15.99			
		-	3	97/97	16.54			
	RG4010 (Δ*csf*)	+	1	99/100	16.64	16.55 ± 1.01	15.35 - 17.75	0.49
		+	2	98/100	17.04			
		+	3	100/101	15.97			

Supplementary Table 9. The use of 16 µM FUdR does not affect N2, *daf-16* and *hsf-1* worm survival fed on wild-type or mutant (RG3603 Δ*bslA*, RG3610 Δ*nos*, RG4010 Δ*csf*) *B. subtilis* cells. Each data set consists of three independent repeats fitted to Boltzmann sigmoid curve and the mean survival time ± SEM and CI are indicated. Independent experimental and control analysis were done side by side and indicated by same number (1, 2, or 3) in the repeat column. Survival data for each treatment was calculated against the correspondent control using Kaplan-Meier test.

COVARIATE	GENE	B	SE	WALD	SIGNIFICANCE
NO	*nos*	0.65113	0.09154	50.5936	<0.0001
CSF	*csf*	0.55610	0.09113	37.2422	<0.0001
BIOFILM	*bslA*	0.99936	0.08236	147.2493	<0.0001
NO/CSF	*nos-csf*	-0.40379	0.15923	6.4305	0.0112
NO/BIOFILM	*nos-bslA*	-0.24010	0.15314	2.4581	0.1169
BIOFILM/CSF	*nos-csf*	-0.21552	0.15280	1.9894	0.1584
NO/CSF/BIOFILM	*nos-csf-bslA*	0.32385	0.25167	1.6559	0.1982

EFFECT OF	HAZARD RATIO EXP(B)	95.0 % CI for LOW to HIGH
BIOFILM DEFICIENCY (Δ*bslA*)	2.45	2.18 to 2.75
NO DEFICIENCY (Δ*nos*)		
when *csf* ACTIVE	1.76	1.52 to 2.03
when *csf* INACTIVE	1.31	1.08 to 1.59
CSF DEFICIENCY (Δ*csf*)		
when *nos* ACTIVE	1.61	1.40 to 1.86
when *nos* INACTIVE	1.20	0.99 to 1.46

Supplementary Table 10. Semiparametric Cox regression analysis[1-3] of *B. subtilis* genes that positively affect *C. elegans* survival. The time until death of worms (n = 300 worms per each lifespan experiment) fed on *B. subtilis*

cells harboring active (present) or inactive (absent) genes promoting worm longevity (covariates, *nos*, *csf*, *bslA*, *nos*-*csf*, *bslA*-*nos*, *csf*-*bslA* and *nos*-*csf*-*bslA*) was analyzed using Cox regression. B is the unstandardized regression coefficient and its standard error (SE), its Wald test significance value (Wald), and the significance determined when the other covariates were set to their mean values. Hazard ratio, Exp(B), for the categorical covariates of interest is the risk of worm death relative to the control case-controlling for other covariates[1-3]. The 95% confidential interval (CI) of the hazard ratio is indicated.

C. elegans strain	Relative to figure	Bacterial strain (genetic background)	Status	Repeats	Number of animals that died/total	Mean survival days	Average of means survival days + S.E.M.	Interval of average survival days with 95 %	Average survival increase (%)
N2	S6	OP50	live	1	50/51	21.22	20.51 + 0.59	19.35 - 21.67	control
				2	30/32	19.25			
				3	27/29	21.16			
			dead	1	45/48	25.16	24.73 + 0.76	23.23 - 26.23	20.58
				2	25/30	24.08			
				3	36/40	24.95			
		NCIB3610	live	1	100/102	29.86	30.10 + 0.99	28.15 - 32.05	control
				2	106/107	30.23			
				3	92/92	30.21			
			dead	1	42/43	25.14	25.79 + 0.8	24.22 - 27.36	- 14.32
				2	30/33	26.12			
				3	25/25	26.11			

Supplementary Table 11. Lifespan analysis related to Supplementary Figure 6.

Only alive *B. subtilis* cells are able to prolong *C. elegans* longevity. Each data set consists of three independent repeats fitted to Boltzmann sigmoid curve and the mean survival time ± SEM and CI are indicated. Percentage (%) survival change of worms fed on dead OP50 *E. coli* cells or dead NCIB3610 *B. subtilis* cells was determined against control worms fed on live OP50 *E. coli* cells or live NCIB3610 *B. subtilis* cells, respectively. Independent experimental and control analysis were done side by side and indicated by same number (1, 2, or 3) in the repeat column. Survival data were calculated by Kaplan-Meier test.

C. elegans strain	Relatived to figure	Bacterial strain (genetic background)	Repeats	Number of animals that died/total	Mean survival days	Average of mean survival days ± S.E.M.	Interval of average survival days with 95 %	Average survival increase (%) / P value
CF1038 (*daf-16*)	6a	OP50 (wt)	1	80/81	13.83	13.87 ± 0.52	12.85 - 14.89	control
			2	96/96	13.88			
			3	90/92	13.90			
		NCIB3610 (wt)	1	77/78	17.17	17.17 ± 0.74	15.72 - 18.62	23.79 / 0.00005
			2	82/82	17.23			
			3	75/81	17.12			
		JH642 (wt)	1	80/80	15.43	15.60 ± 0.68	14.27 - 16.93	12.47 / 0.098
			2	80/84	15.70			
			3	105/114	15.68			
PS3551 (*hsf-1*)	6b	OP50	1	80/80	10.86	11.97 ± 0.58	10.83 - 13.11	control
			2	82/82	12.05			
			3	90/90	13.00			
		NCIB3610	1	80/80	17.95	16.25 ± 0.60	15.08 - 17.42	35.76 / 0
			2	70/70	16.32			
			3	76/78	14.48			
		JH642	1	84/84	14.17	13.85 ± 0.56	12.76 - 14.94	15.71 / 0.0011
			2	80/80	13.71			
			3	85/86	13.67			

Supplementary Table 12. Lifespan analysis related to Figure 6.
The transcription factors DAF-16 and HSF-1 are necessary for the prolongevity effect of *B. subtilis* on *C. elegans*. Each data set consists of three independent repeats fitted to Boltzmann sigmoid curve and the mean survival time ±SEM and CI are indicated. Percentage (%) survival change of wild-type (N2), *daf-16* (CF1038) or *hsf-1* (PS3551) worms fed on wild-type domesticated (JH642) or undomesticated (NCIB3610) *B. subtilis* cells was determined against worms (wild-type, *daf-16* or *hsf-1*) fed on control OP50 *E. coli* cells. Independent experimental and control analysis were done side by side and indicated by same number (1, 2, or 3) in the repeat column. P-values for each treatment were calculated against the correspondent control by Log-rank test. Survival data were calculated using Kaplan-Meier test.

C. elegans strain	Relatived to figure	Bacterial strain (genetic background)	Repeats	Number of animals that died/total	Mean survival days	Average of mean survival days ± S.E.M.	Interval of average survival days with 95 %	Average survival increase (%) / P value
N2	7a	NCIB3610 (wt)	1	100/102	29.86	30.10 ± 0.99	28.15 - 32.05	control
			2	106/107	30.23			
			3	92/92	30.21			
		RG3603 (Δ*bslA*)	1	93/94	23.16	22.17 ± 0.56	21.07 - 23.27	- 26.35 / 0
			2	95/95	22.00			
			3	96/96	21.35			
		RG3610 (Δ*nos*)	1	86/87	26.08	26.33 ± 0.90	24.57 - 28.09	- 12.52 / 0.0016
			2	95/95	26.48			
			3	86/87	26.43			
		RG4010 (Δ*csf*)	1	100/100	26.34	26.80 ± 0.94	24.96 - 28.64	- 10.96 / 0.0037
			2	100/100	26.98			
			3	105/102	27.08			
CF1038 (*daf-16*)	7b	NCIB3610	1	98/100	17.89	17.55 ± 0.74	16.48 - 18.62	control
			2	94/100	17.34			
			3	97/101	17.42			
		RG3603	1	86/99	13.95	14.91 ± 0.44	14.04 - 15.78	- 15.04 / 0.00000002
			2	80/99	14.65			
			3	100/100	16.13			
		RG3610	1	99/99	15.89	15.85 ± 0.5	14.67 - 17.03	- 9.69 / 0.307
			2	98/109	15.38			
			3	100/100	16.28			
		RG4010	1	96/99	15.68	16.37 ± 0.59	15.20 - 17.54	- 6.72 / 0.545
			2	100/100	17.85			
			3	100/101	15.58			
PS3551 (*hsf-1*)	7c	NCIB3610	1	100/100	17.44	17.35 ± 0.50	16.18 - 18.52	control
			2	100/101	16.05			
			3	97/99	18.56			
		RG3603	1	100/102	15.18	14.72 ± 0.55	13.65 - 15.79	- 15,16 / 0.038
			2	97/100	14.69			
			3	100/100	14.29			
		RG3610	1	100/106	18.49	16.21 ± 0.63	14.98 - 17.44	- 6.57 / 0.1907
			2	95/100	15.94			
			3	102/102	15.20			
		RG4010	1	99/100	16.64	16.55 ± 0.61	15.35 - 17.75	- 4.61 / 0.3124
			2	98/100	17.04			
			3	100/101	15.97			

Supplementary Table 13. Lifespan analysis related to Figure 7.
Each *B. subtilis* prolongevity property (NO/CSF production and biofilm formation proficiency) requires DAF-16 and HSF-1 functionality. The showed data set consists of three independent repeats fitted to Boltzmann sigmoid curve and the mean survival time ± SEM, *p* -value and CI are indicated. Percentage (%) survival change of wild-type (N2), *daf-16* (CF1038) or *hsf-1* (PS3551) worms fed on the different *B. subtilis* mutant cells (Δ*bslA* , Δ*nos* , or Δ*csf*) was determined against worms (wild-type, *daf-16* or *hsf-1*) fed on control NCIB3610 (wild-type) *B. subtilis* cells. Independent experimental and control analysis were done side by side and indicated by same number (1, 2, or 3) in the repeat column. *P* -values for each treatment were calculated against the correspondent control by Log-rank test. Survival data were calculated using Kaplan-Meier test.

C. elegans strain	Relatived to figure	Bacterial strain (genetic background)	Repeats	Number of animals that died/total	Mean survival days	Average of mean survival days ± S.E.M.	Interval of average survival days with 95 %	Average survival increase (%) / P value
CB1370 (*daf-2* / e1370)	8a	OP50 (wt)	1	79/80	32.7	31.82 ± 1.68	28.84 -35.10	control
			2	97/98	31.37			
			3	100/106	31.38			
		NCIB3610 (wt)	1	74/74	37.62	34.84 ± 1.81	31.26 - 38.39	9.49 / 0.663
			2	77/78	37.74			
			3	97/97	32.16			

Supplementary Table 14. Lifespan analysis related to Figure 8.
The prolongevity effect of *B. subtilis* on worm survival requires an active insulin-like signaling pathway. *daf-2* (CB1370) worms were grown at 15 ºC with OP50 *E. cells* as food source until worms reached late L4/young adult stage. At this developmental time worms were taken, washed and shifted to 20 ºC in the presence of OP50 *E. coli* cells or NCIB3610 *B. subtilis* cells as new food sources. The average survival increase was determined considering worms fed on OP50 *E. coli* cells as control. Each data set consists of three independent repeats fitted to Boltzmann sigmoid curve and the mean survival time ± SEM, *p* -value and CI are indicated. Independent experimental and control analysis were done side by side and indicated by same number (1, 2, or 3) in the repeat column. *P* -values for each treatment were calculated against the correspondent control by Log-rank test. Survival data were calculated using Kaplan-Meier test.
